# Supplementary material for: Clonal reconstruction from co-occurrence of vector integration sites accurately quantifies expanding clones in vivo
Source: Nat Commun. 2022 Jun 28;13:3712. doi: 10.1038/s41467-022-31292-6 (PMC9240075; doi:10.1038/s41467-022-31292-6)
Supplement: Supplementary file 1 — Supplementary Information [file 41467_2022_31292_MOESM1_ESM.pdf]

Supplementary Figures, Tables and Notes

for

Clonal reconstruction from co-occurrence of vector integration sites accurately quantifies expanding clones in vivo

Sebastian Wagner, Christoph Baldow, Andrea Calabria, Laura Rudilosso, Pierangela Gallina, Eugenio Montini, Daniela Cesana, Ingmar Glauche

Supplementary Figure S1

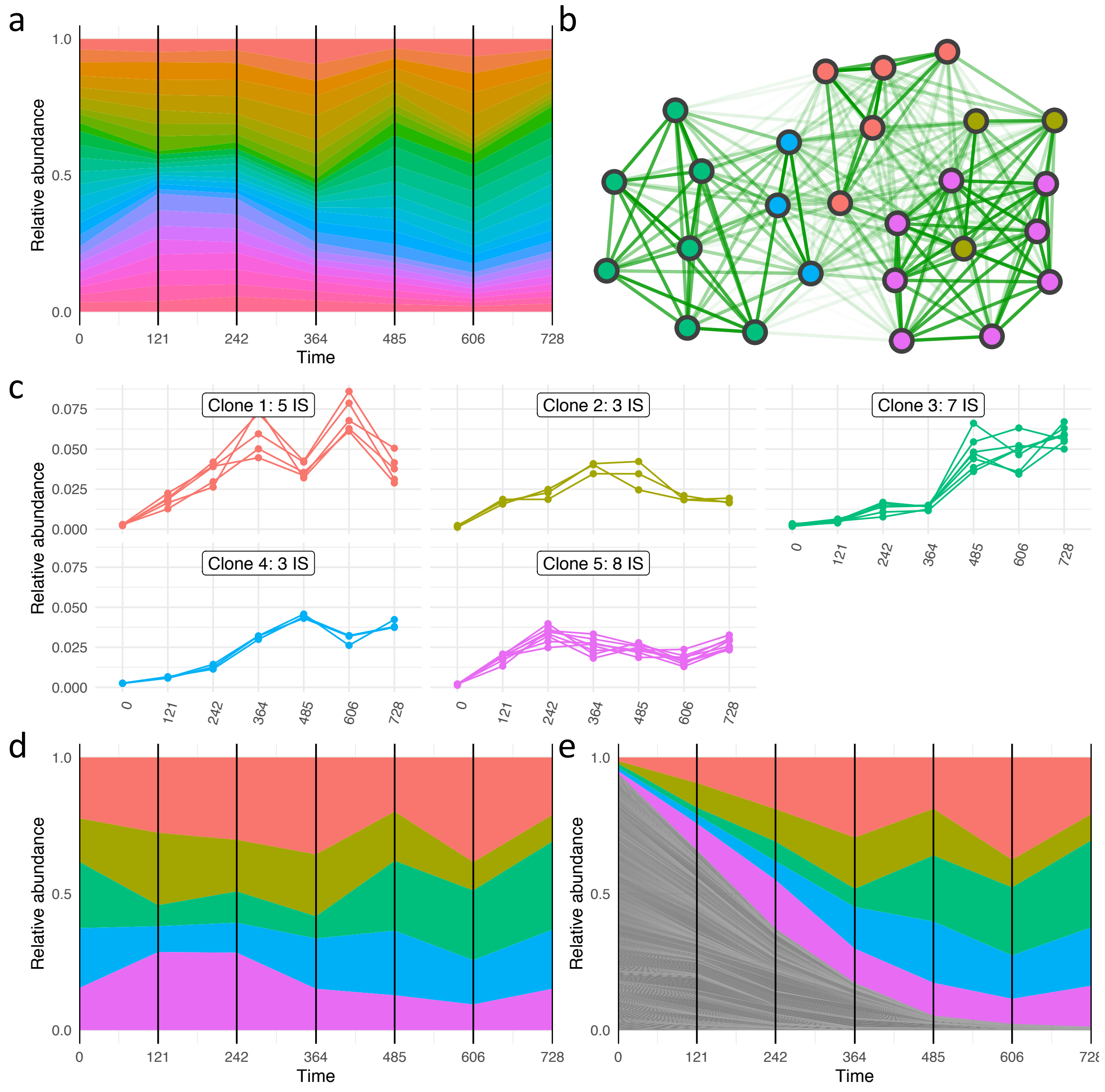

Supplementary Figure S1: Reconstruction for a simulated time course with low measurement noise σ = 0.15.

Time courses are initialized with 100 clones, each with 100 identical cells. The number of IS per clone are chosen from a Poisson distribution with average number of IS λ=5. **a** relative abundances of IS as a function of time after applying the filtering step. **b** The similarity (indicated by edge brightness) between each pair of integration sites is superimposed by the optimal clustering (indicated by color of the nodes) obtained from the reconstruction pipeline. **c** time series of all IS assigned to the same clusters/clones (color coding corresponds to subfigure B). **d** corrected clonal time series for the five identified clones. **e** shows the corrected clonal time course together with the IS that did not pass the initial filtering step (grey).

Supplementary Figure S2

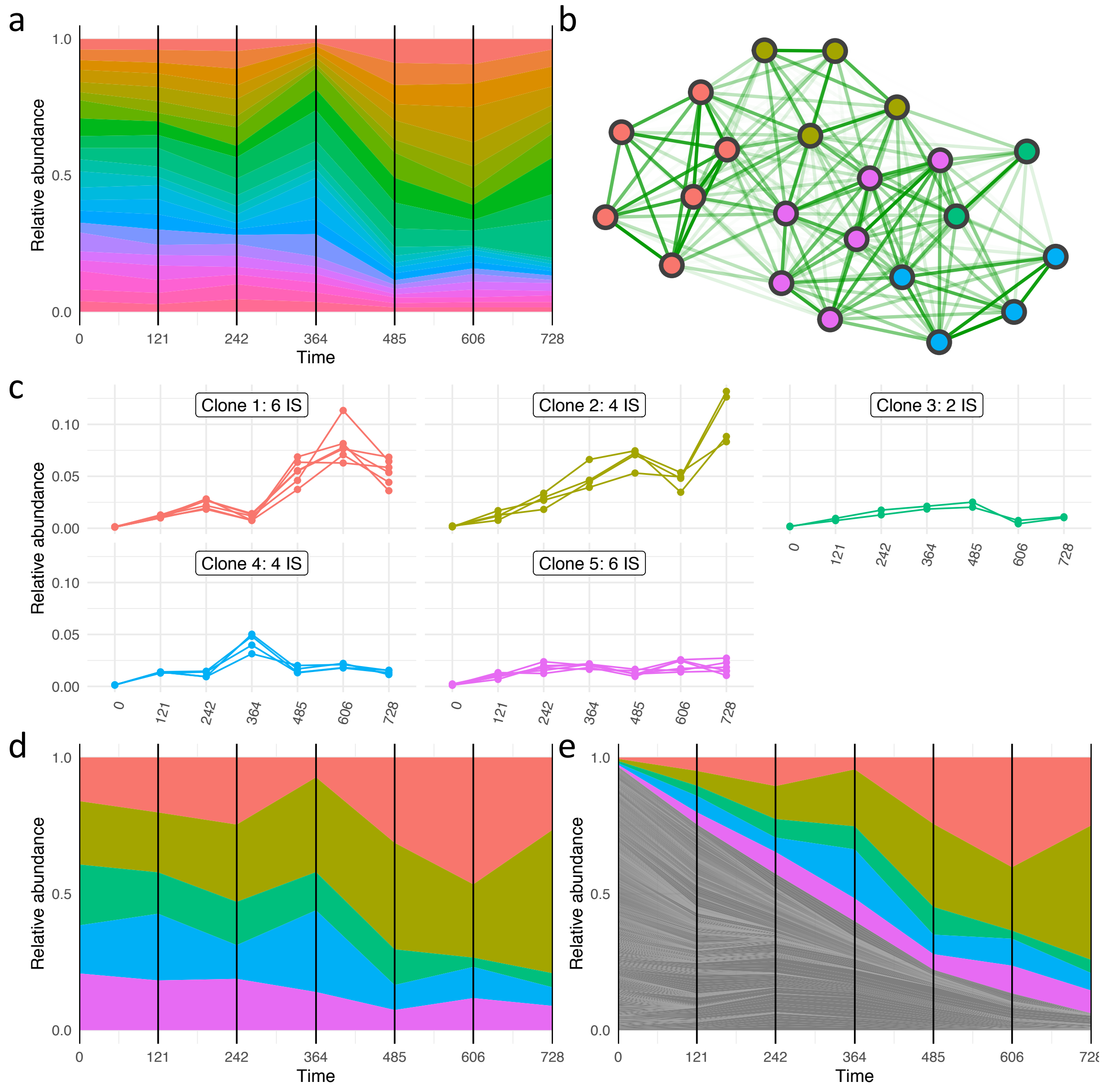

Supplementary Figure S2: Reconstruction for a simulated time course with high measurement noise σ = 0.25.

Time courses are initialized with 100 clones, each with 100 identical cells. The number of IS per clone are chosen from a Poisson distribution with average number of IS λ=5. **a** relative abundances of IS as a function of time after applying the filtering step. **b** The similarity (indicated by edge brightness) between each pair of integration sites is superimposed by the optimal clustering (indicated by color of the nodes) obtained from the reconstruction pipeline. **c** time series of all IS assigned to the same clusters/clones (color coding corresponds to subfigure B). **d** corrected clonal time series for the five identified clones. **e** shows the corrected clonal time course together with the IS that did not pass the initial filtering step (grey).

Supplementary Figure S3

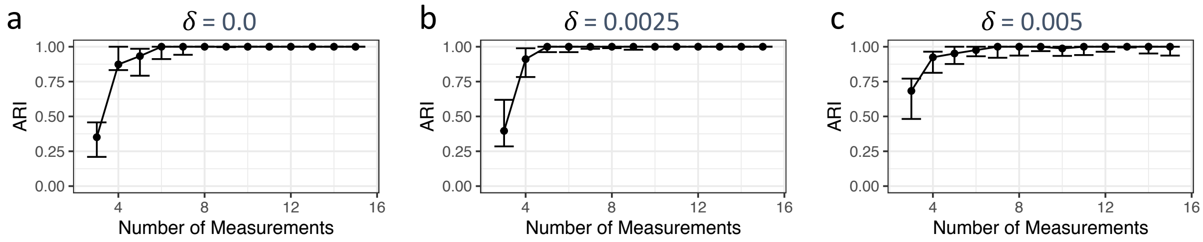

Supplementary Figure S3: Clonal reconstruction for different reference scenarios

The figure complements Figure 3 in the main text, here reporting the quality of the clonal reconstruction process for the scenario that not 100 clones, but 1000 clones are initialized, each containing 10 cells with identical features. According to the columns in Figure 3 the differentiation rate δ increases from subfigure A to C to mimic different tendencies for clonal conversion. The quantitative analysis is based on 20 independent simulations each (points indicate the median; whiskers correspond to the first and third quartile).

Supplementary Figure S4

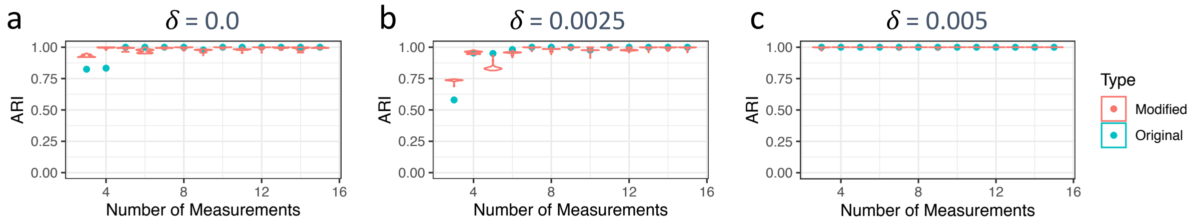

Supplementary Figure S4: Clonal reconstruction for indistinguishable IS in different clones

The figure complements Figure 3 in the main text, here reporting the reconstruction quality for the unlikely case that one IS of the finally dominating clone is also found in an indistinguishable location in a different clone. While the green dots reflect the adjusted Rand index (ARI) for the particular simulations in Figure 3 a to i, the violin plots correspond to modified time courses, in which one of the IS of the largest clones is shared with a randomly chosen other IS. Based on this modified time course we rerun the reconstruction pipeline and repeat the choice of the “sibling IS” 100 times. The distribution of obtained ARI values for the 100 repetitions is shown by red violin plots.

Supplementary Figure S5

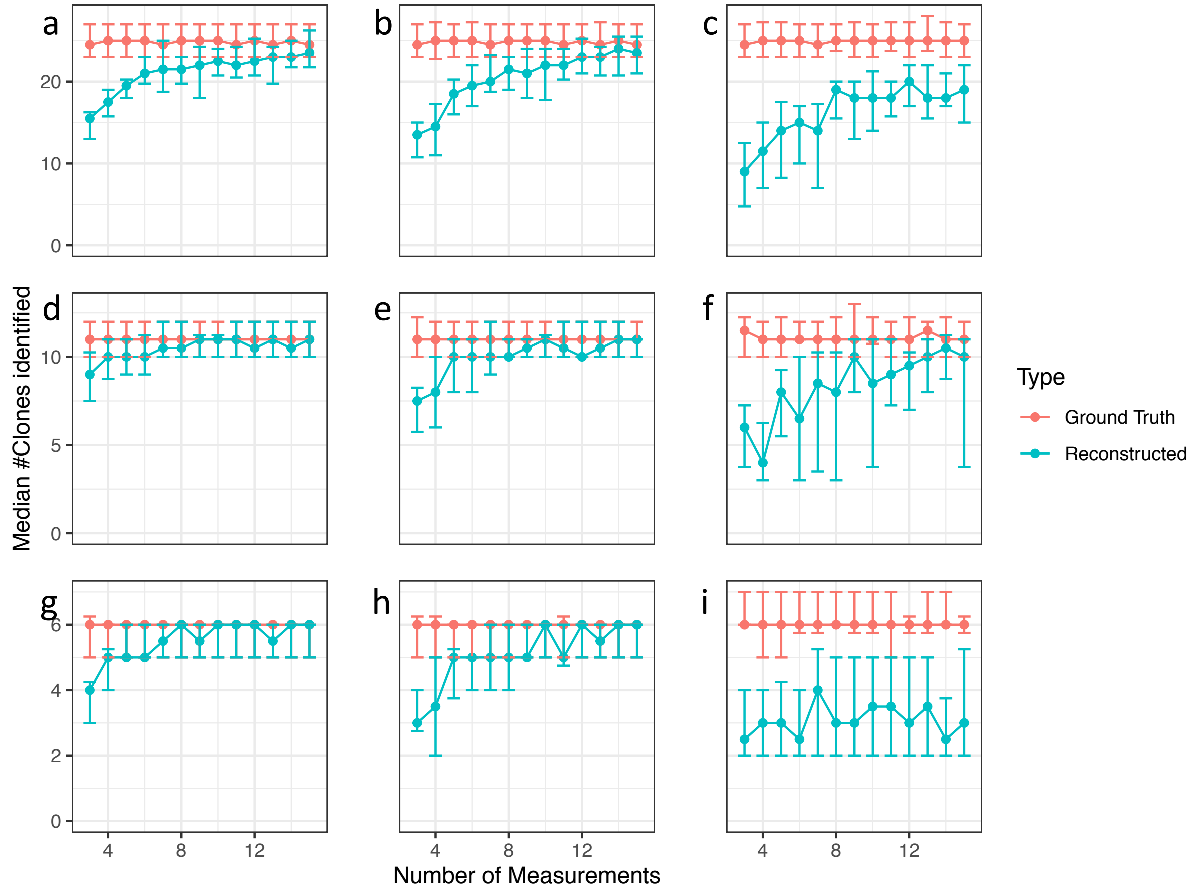

Supplementary Figure S5: Fraction of reconstructed clones

The figure complements Figure 4 in the main text, here reporting the number of reconstructed clones (green) compared to the true number of clones (red) in the respective subset of IS that remained after the filtering step. Rows refer to the average number of IS $\lambda = 2$ (**a,b,c**), $\lambda = 5$ (**d,e,f**) and $\lambda = 10$ (**g,h,i**). The column represents increasing levels of the measurement noise $\sigma = 0.025$ (**a,d,g**), $\sigma = 0.04$ (**b,e,h**) and $\sigma = 0.08$ (**c,f,i**). Each data point is based on 20 independent simulation runs (points indicate the median adjusted Rand index (ARI); whiskers correspond to the first and third quartile) for the differentiation rate $\delta = 0.0025$.

Supplementary Figure S6

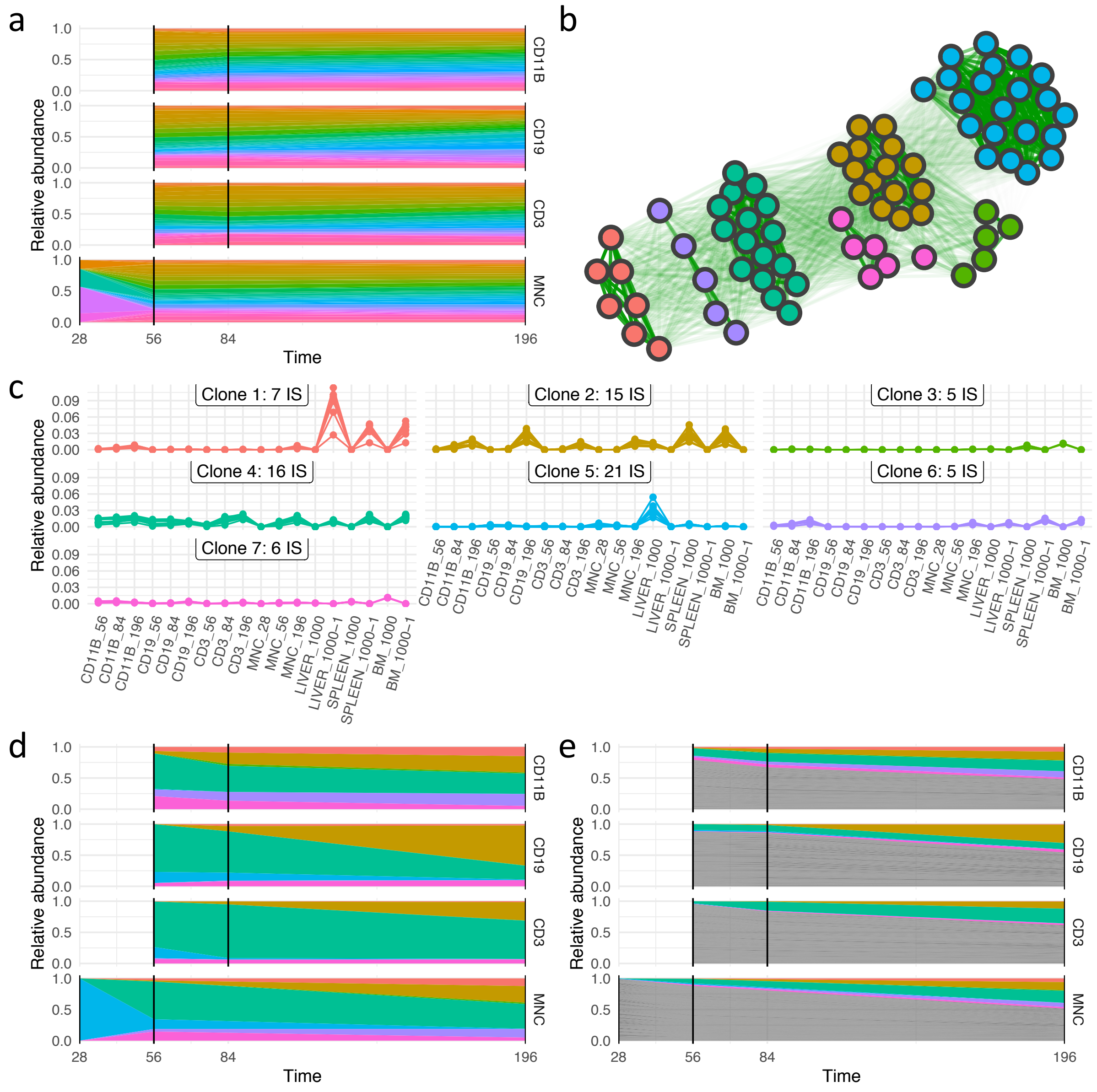

Supplementary Figure S6: Experimental data and clonal reconstruction for mouse pool E3D.

Mice were transplanted with HSPCs previously transduced with a neutral SINLV-based vector. Collected cells were sorted according to their immune phenotype and analyzed separately for IS abundance.

a relative abundances of IS as a function of time for CD11b, CD19, CD3, and mononuclear cells, for which multiple measurements are available. **b** similarity (indicated by edge brightness) between each pair of integration sites superimposed by the optimal clustering (indicated by color of the nodes) obtained from the reconstruction pipeline. **c** time series of all IS assigned to the same clusters/clones (color coding corresponds to subfigure B). **d** shows the corrected clonal time course for the seven identified clones. **e** shows the corrected clonal time series together with the IS that did not pass the initial filtering step (grey).

Supplementary Figure S7

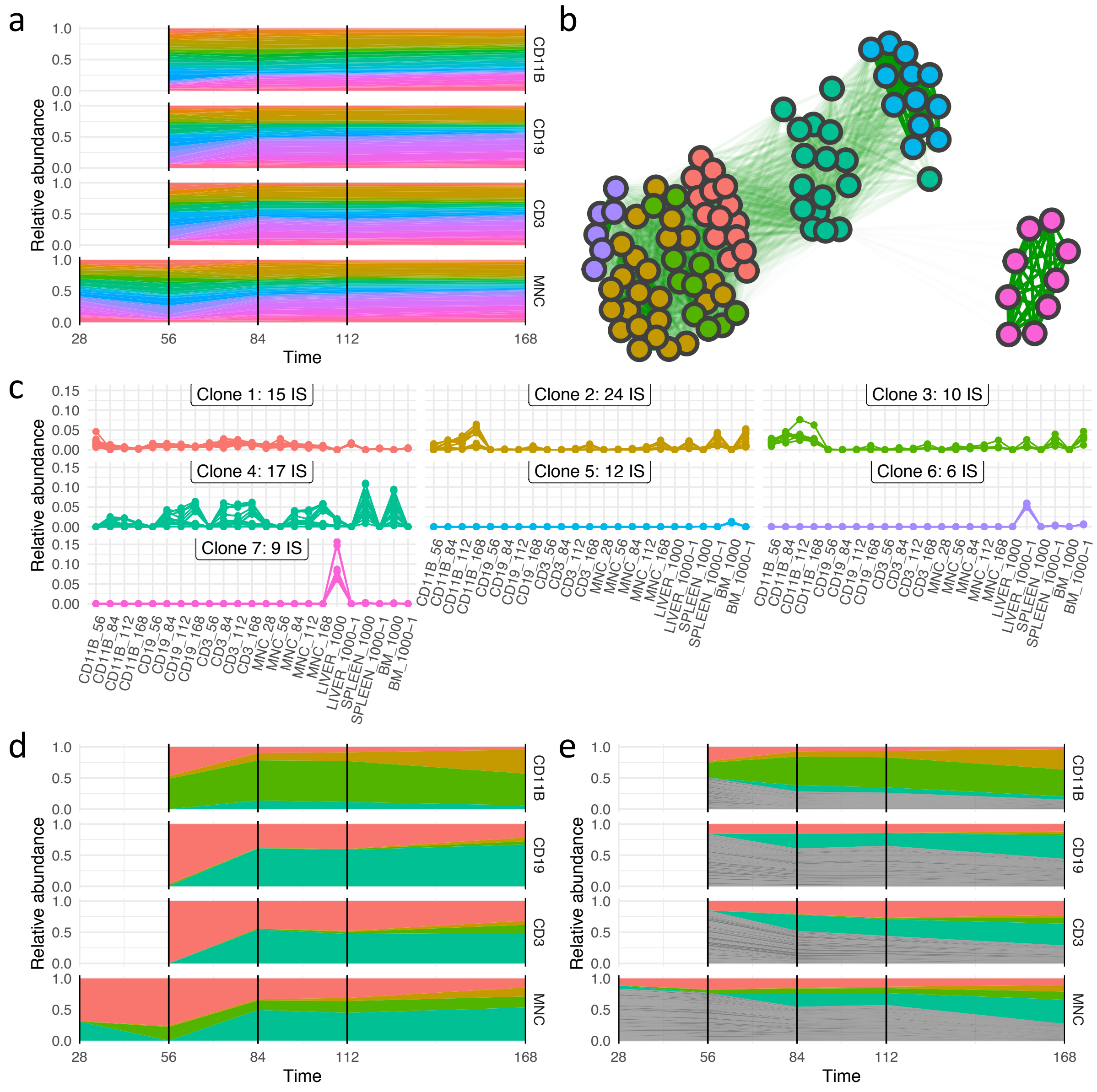

Supplementary Figure S7: Experimental data and clonal reconstruction for mouse pool E2L.

Mice were transplanted with HSPCs previously transduced with a neutral SINLV-based vector. Collected cells were sorted according to their immune phenotype and analyzed separately for IS abundance.

a relative abundances of IS as a function of time for CD11b, CD19, CD3, and mononuclear cells, for which multiple measurements are available. **b** similarity (indicated by edge brightness) between each pair of integration sites superimposed by the optimal clustering (indicated by color of the nodes) obtained from the reconstruction pipeline. **c** time series of all IS assigned to the same clusters/clones (color coding corresponds to subfigure B). **d** shows the corrected clonal time course for the seven identified clones. **e** shows the corrected clonal time series together with the IS that did not pass the initial filtering step (grey).

Supplementary Figure S8

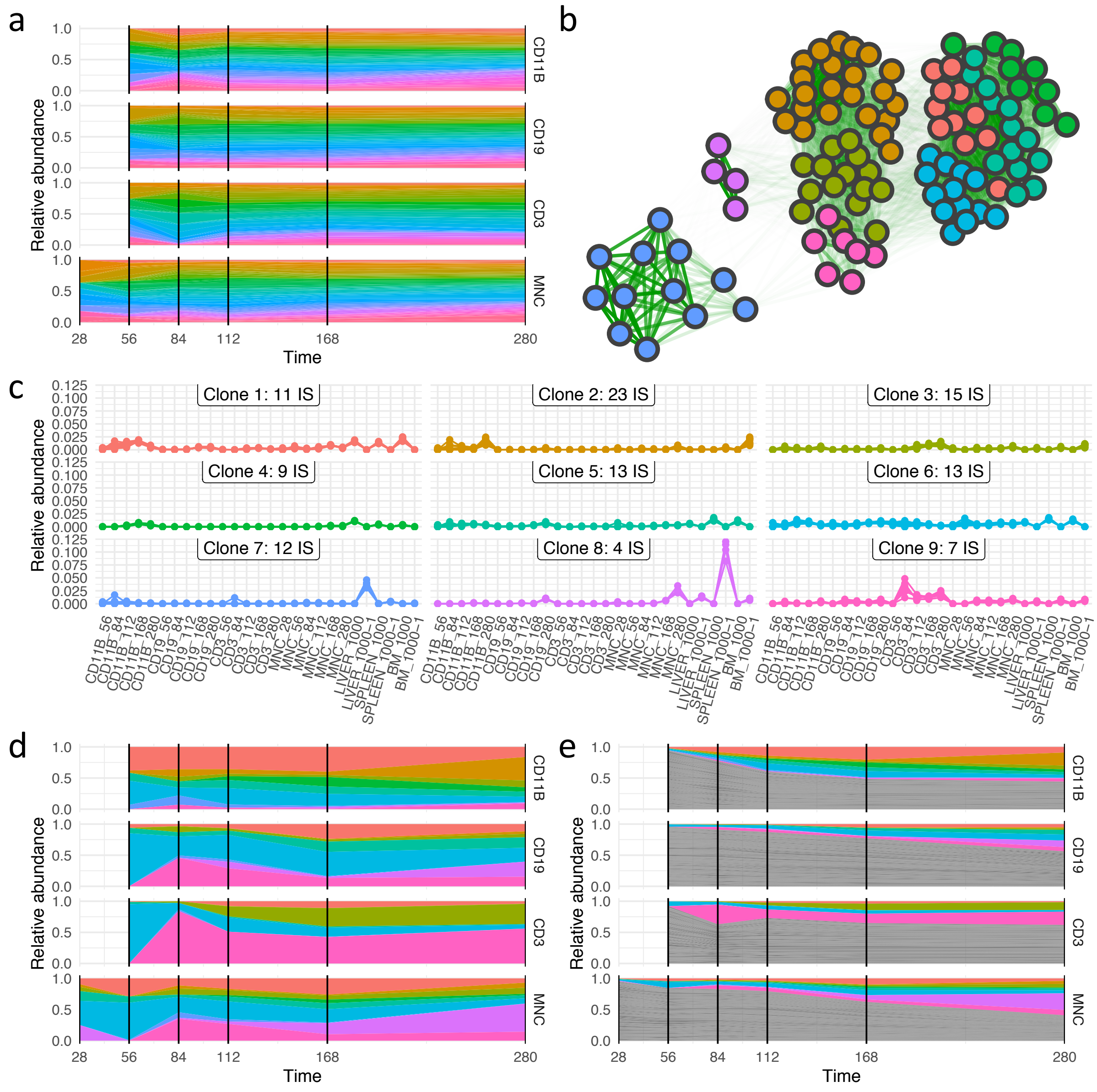

Supplementary Figure S8: Experimental data and clonal reconstruction for mouse pool E2I.

Mice were transplanted with HSPCs previously transduced with a neutral SINLV -based vector. Collected cells were sorted according to their immune phenotype and analyzed separately for IS abundance.

a relative abundances of IS as a function of time for CD11b, CD19, CD3, and mononuclear cells, for which multiple measurements are available. **b** similarity (indicated by edge brightness) between each pair of integration sites superimposed by the optimal clustering (indicated by color of the nodes) obtained from the reconstruction pipeline. **c** time series of all IS assigned to the same clusters/clones (color coding corresponds to subfigure B). **d** shows the corrected clonal time course for the seven identified clones. **e** shows the corrected clonal time series together with the IS that did not pass the initial filtering step (grey).

Supplementary Figure S9

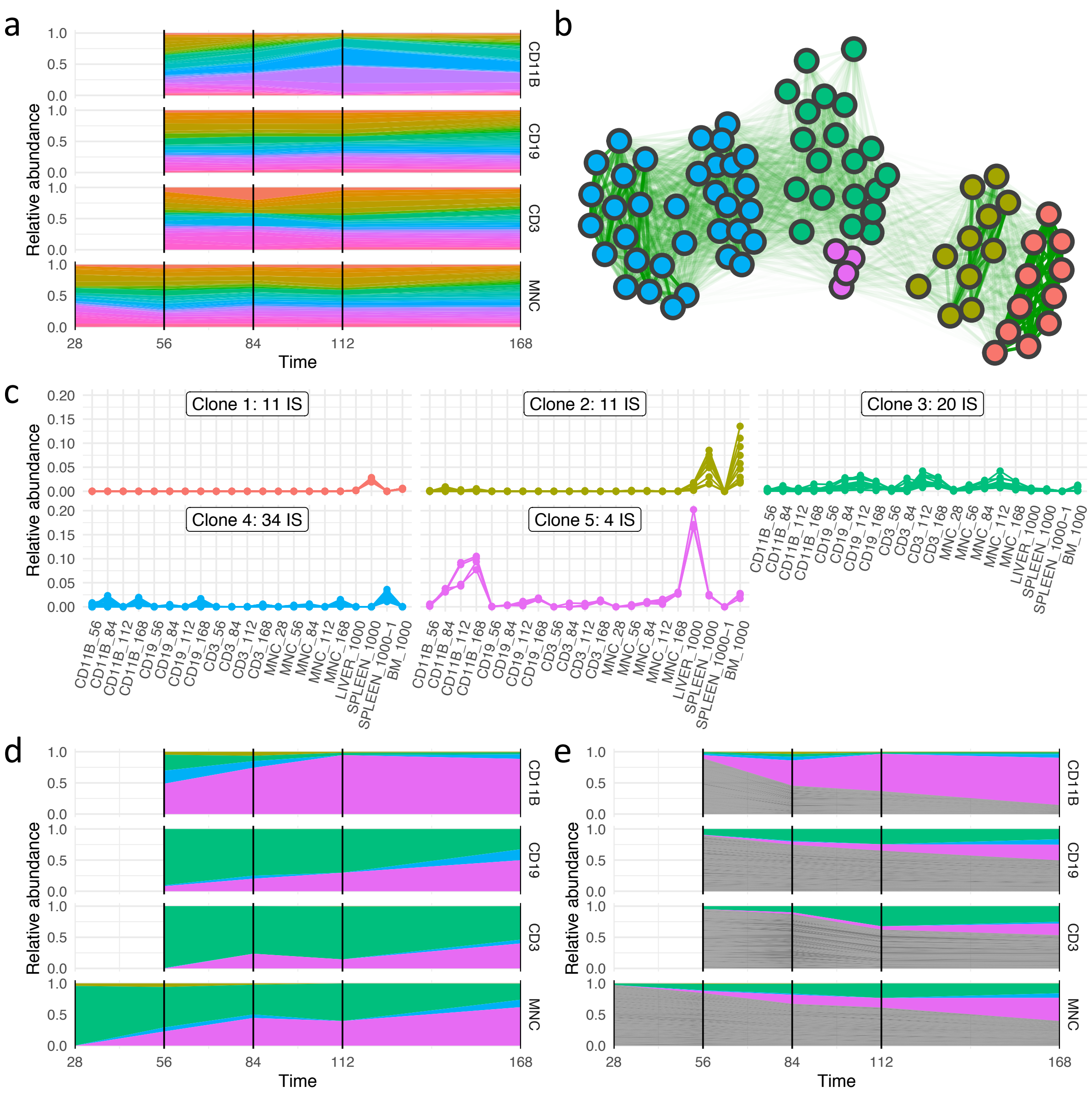

Supplementary Figure S9: Experimental data and clonal reconstruction for mouse pool E2G.

Mice were transplanted with HSPCs previously transduced with a neutral SINLV -based vector. Collected cells were sorted according to their immune phenotype and analyzed separately for IS abundance.

a relative abundances of IS as a function of time for CD11b, CD19, CD3, and mononuclear cells, for which multiple measurements are available. **b** similarity (indicated by edge brightness) between each pair of integration sites superimposed by the optimal clustering (indicated by color of the nodes) obtained from the reconstruction pipeline. **c** time series of all IS assigned to the same clusters/clones (color coding corresponds to subfigure B). **d** shows the corrected clonal time course for the five identified clones. **e** shows the corrected clonal time series together with the IS that did not pass the initial filtering step (grey).

Supplementary Figure S10

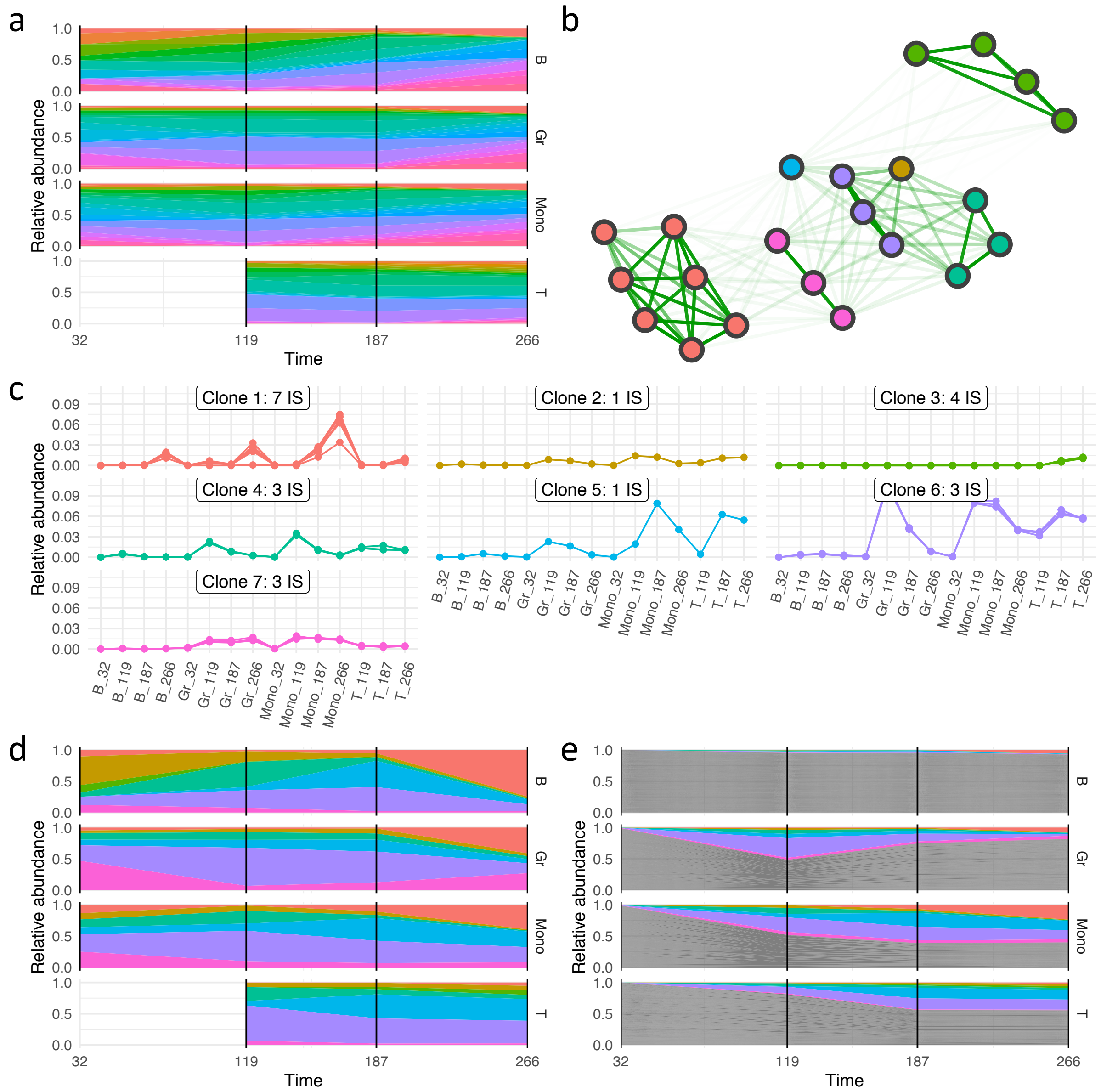

Supplementary Figure S10: Experimental data and clonal reconstruction for a rhesus macaques at time point 266 d.

The analysis follows the same scheme as Figure 7 in the main text, although all time points beyond day 266 are neglected. The dominant “red” clone is already identified at this early time point, although an additional, seventh IS is associated with it.

a relative abundances of IS as a function of time for the four subcompartments, for which multiple measurements are available. **b** similarity (indicated by edge brightness) between each pair of IS superimposed by the optimal clustering (indicated by color of the nodes) obtained from the reconstruction pipeline. **c** time series of all IS assigned to the same clusters/clones (color coding corresponds to subfigure B). **d** shows the corrected clonal time course for the seven identified clones. **e** shows the corrected clonal time series together with the IS that did not pass the initial filtering step (grey).

Supplementary Figure S11

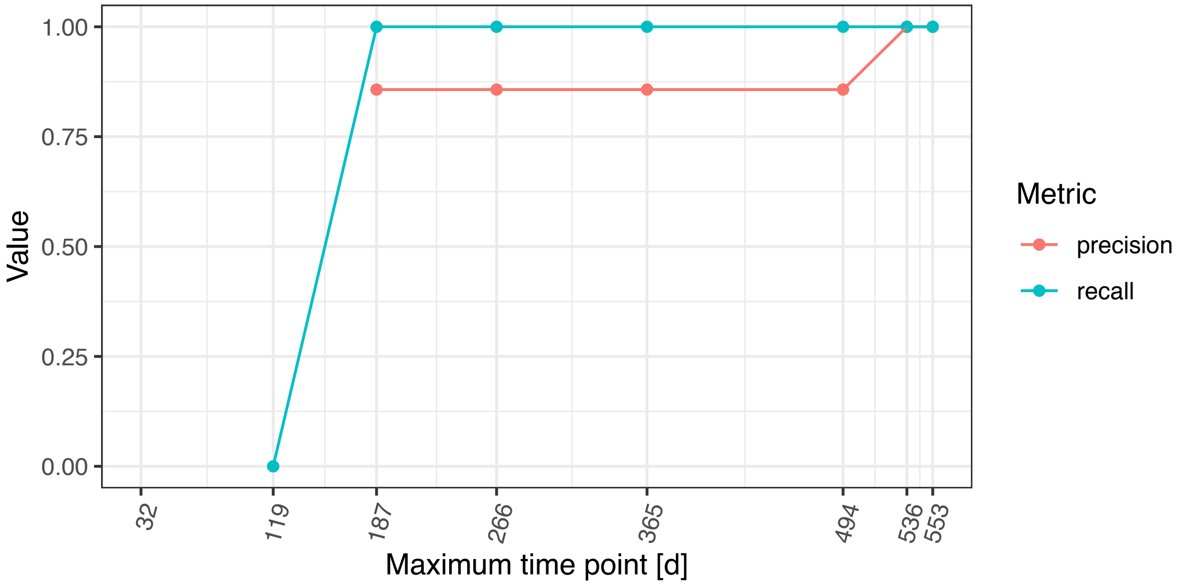

Supplementary Figure S11: Precision and recall for the dominating clone in a rhesus macaques as a function of time.

We know from the experimental results (Espinoza et al., Molecular Therapy, 2019) and our own analysis (Figure 7) that six continuously detectable IS can be mapped to the single expanding clone. Supplementary Figure S10 indicates that this association is already feasible at an earlier time point. We report precision and recall for the identification of the IS within this clone based on maximum time point available. This mimics the continuous monitoring approach applied in GT applications in which all previous samples are available for clonal analysis. The early rise in precision and recall indicates that already with three measurement time points in four different compartments, there is enough signal to identify the association of IS jointly occurring in the dominating clone.

Supplementary Figure S12

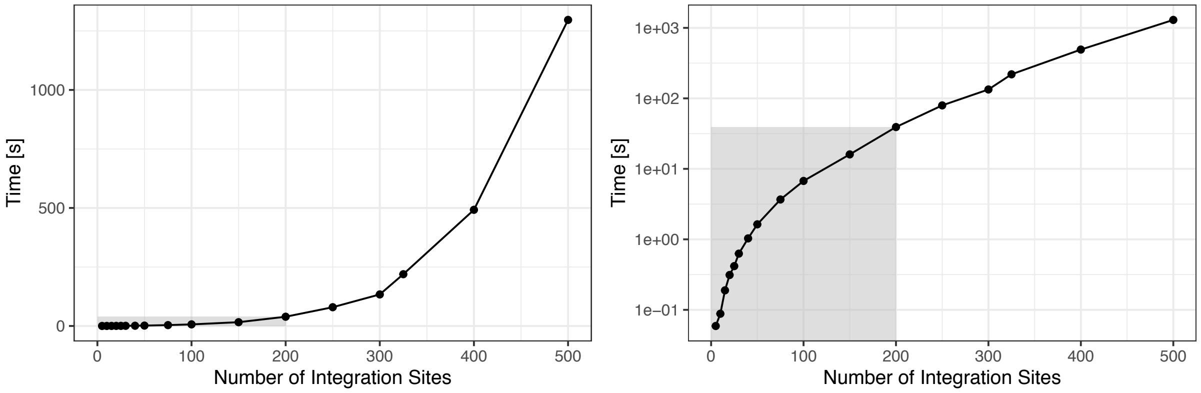

Supplementary Figure S12: Performance test and scaling of our computational approach.

We compared the running times for random input samples containing different numbers of IS measured at 20 independent time points. The results are shown in a linear and a logarithmic representation. As we outlined, it is sensible to limit the amount of considered IS, not because of computational constraints but to avoid spurious correlations resulting from an abundance of IS measurements close to the detection limit. Considering a detection limit between 0.1% and 1% we did not encounter cases in which more than 200 IS were analyzed. We marked this region in grey to indicate that corresponding simulations can be performed in the range of a few seconds.

Supplementary Figure S13

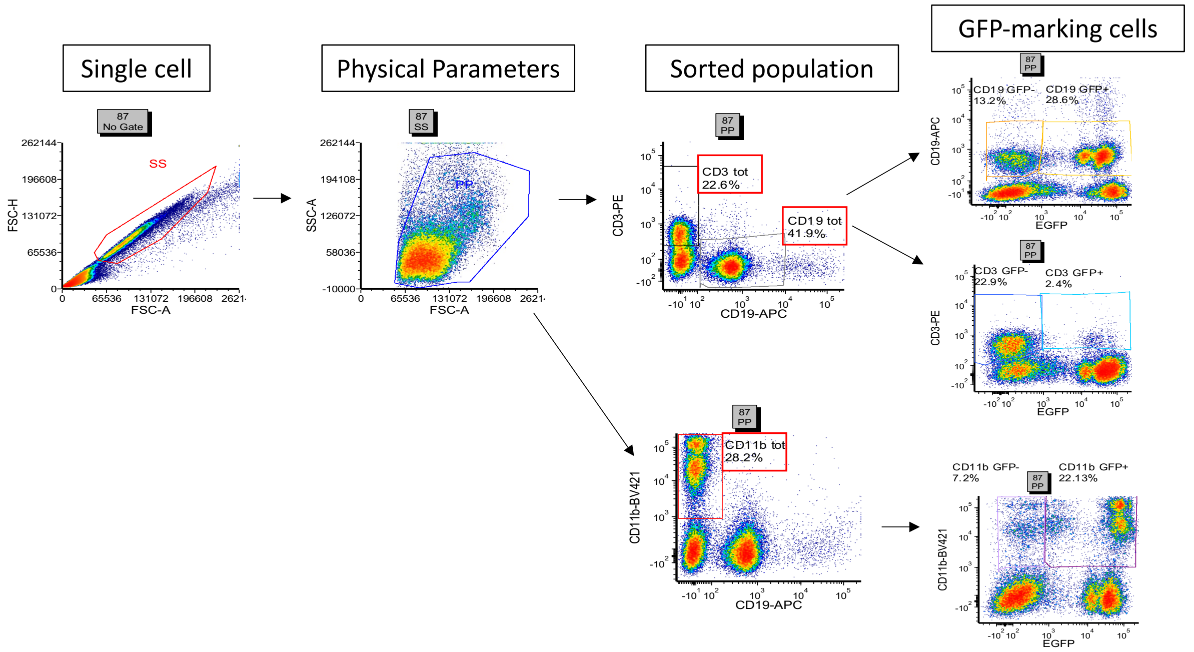

Supplementary Figure S13: Gating strategy for cell sorting.

Dot plots demonstrating the sequential gating process used for the selection of the sorted lineage positive cell, T-cells (CD3+), B cells (CD19+) and Myeloid cells (CD11b+). Briefly, single cells were selected by representing FSC Height/Weight parameter vs Area. Then, cell populations were selected based on their FSC-A/SSC-A parameter. Finally, cells expressing the mentioned markers were gated based on the respective negative controls of unstained cells. Within each cell subsets, we checked the % of double positive GFP+ cells, reflecting the overall transduction level.

Supplementary Table S1: Relative composition of the different mixes for the validation assay. JY indicating a polyclonal background.

Volume	Mix 1	Mix 2	Mix 3	Mix 4	Mix 5	Mix 6	Mix 7
JY	0.60	0.35	0.10	0.09	0.07	0.24	0.56
ID#27	0.05	0.05	0.05	0.40	0.03	0.08	0.25
ID#30	0.25	0.50	0.75	0.03	0.40	0.25	0.08
ID#37	0.05	0.05	0.05	0.08	0.25	0.40	0.03
ID#46	0.05	0.05	0.05	0.40	0.25	0.03	0.08

Supplementary Table S2: Genomic position of clone specific IS for the K562 cell clones

Clone ID (a)	N IS (b)	Chr (c)	Integration_locus (d)	Gene Name (e)
ID#27	1	5	72866480	UTP15
ID#30	4	2	223461674	FARSB
		14	97342533	VRK1
		2	208325826	CREB1
		4	129335149	LOC100507487
ID#37	6	8	91674211	TMEM64
		18	38162304	LINC01477
		14	81652945	GTF2A1
		12	96398298	LTA4H
		X	120619080	MIR3672
		7	110789918	IMMP2L
ID#46	10	6	45172433	SUPT3H
		19	8002970	TIMM44
		4	909971	GAK
		11	8711542	RPL27A
		1	160414727	VANGL2
		4	120428505	PDE5A
		4	63782063	ADGRL3-AS1
		15	78770928	IREB2
		20	33384442	NCOA6
		6	47118860	TNFRSF21

(a) **Clone ID:** identifier of the K562 clone
(b) **N IS:** number of different LV integrations identified
(c) **Chr:** chromosome number of the targeted gene in the human genome
(d) **Integration locus:** genomic coordinate of the integration point
(e) **Gene Symbol:** gene symbol of the targeted gene

Supplementary Table S3: Expected and reconstructed clonal abundances for the validation assay (compare Figure 5). Red numbers indicate divergence between reconstructed and expected.

		Mix 1	Mix 2	Mix 3	Mix 4	Mix 5	Mix 6	Mix 7
**Expectation** (rel. abundance without background)	ID#27	0.12	0.08	0.06	0.44	0.03	0.11	0.57
	ID#30	0.62	0.77	0.83	0.03	0.43	0.33	0.18
	ID#37	0.12	0.08	0.06	0.09	0.27	0.53	0.07
	ID#46	0.12	0.08	0.06	0.44	0.27	0.04	0.18
**Reconstruction** (naïve, ID#27 wrongly assigned to ID#46)	~~ID#27~~	0.00 (-0.12)	0.00 (-0.08)	0.00 (-0.06)	0.00 (-0.44)	0.00 (-0.03)	0.00 (-0.11)	0.00 (-0.57)
	ID#30	0.74 (+0.12)	0.86 (+0.09)	0.90 (+0.06)	0.07 (+0.03)	0.46 (+0.03)	0.36 (+0.03)	0.39 (+0.20)
	ID#37	0.12 (-0.00)	0.07 (-0.01)	0.05 (-0.00)	0.14 (+0.05)	0.27 (-0.00)	0.55 (+0.03)	0.12 (+0.05)
	ID#46	0.14 (+0.01)	0.07 (-0.00)	0.05 (-0.00)	0.79 (+0.36)	0.27 (+0.00)	0.09 (+0.05)	0.49 (+0.31)
**Reconstruction** (corrected, reconstruction of four clones)	ID#27	0.15 (+0.03)	0.10 (+0.02)	0.07 (+0.01)	0.53 (+0.09)	0.04 (+0.01)	0.14 (+0.03)	0.64 (+0.07)
	ID#30	0.63 (+0.00)	0.78 (+0.01)	0.84 (+0.00)	0.03 (-0.00)	0.43 (+0.00)	0.31 (-0.01)	0.16 (-0.03)
	ID#37	0.10 (-0.02)	0.06 (-0.01)	0.05 (-0.01)	0.07 (-0.02)	0.25 (-0.02)	0.48 (-0.05)	0.05 (-0.02)
	ID#46	0.11 (-0.01)	0.06 (-0.01)	0.05 (-0.01)	0.37 (-0.07)	0.27 (+0.00)	0.07 (+0.03)	0.16 (-0.03)

Supplementary Table S4: Simulation Parameters

	Figure 3, S4	Figure 4, S5	Figure S1	Figure S2	Figure S3
number of clones	100	100	100	100	1000
cells per clone	100	100	100	100	10
number of measurements	3 ... 15	3 ... 15	7	7	3 ... 15
average number of IS λ	5	2, 5, 10	5	5	5
clonal variability ν	0	0, 0.125, 0.25, 0.375, 0.5	0.25	0.25	0
measurement noise σ	0.025	0.025, 0.04 0.08	0.15	0.25	0.025
proliferation rate p_{max}	0.06	0.06	0.06	0.06	0.06
carrying capacity K	10,000	10,000	10,000	10,000	10,000
differentiation rate d	0.04	0.04	0.04	0.04	0.04
standard deviation of the differentiation rate δ	0.0, 0.0025, 0.005	0.0025	0.01	0.01	0.0, 0.0025, 0.005

Supplementary Table S5: Vector copy numbers of experimental samples from the mouse experiments compared to the values obtained from the reconstruction process.

We report VCN for the different grafts as well as mean VCN (plus 95% CI) for blood and tissue samples (n = 12 to 18 per pool), all measured by ddPCR. We also provide the average number of IS obtained from the clonal reconstruction of *MultIS* that focusses on the most abundant IS only.

Mouse Pool	VCN graft	Mean VCN samples [95% CI]	Average number of IS per clone reconstructed by *MultIS*
E2G	13.59	8.93 [6.55, 11.30]	16.00
E2I	13.59	8.89 [6.43, 11.34]	11.89
E2L	13.59	6.96 [5.22, 8.70]	13.29
E3D	13.42	12.70 [10.41, 14.99]	10.71
E4C	16.27	12.78 [10.34, 15.22]	6.62

Supplementary Notes

Supplementary Note 1: Technical description of the reconstruction pipeline

Similarity of IS abundances

We applied an R^2 approach to quantify the similarity in the abundance of two IS, which is based on the *relative* IS reads $I_i^{*rel}(t) := \frac{I_i(t)}{\sum_l I_l(t)}$, either obtained from simulated or measured data. Comparing the $I_i^{*rel}(t)$ for all possible pairs of IS *i, j* retained after filtering and calculating a linear regression through the origin (compare Figure 2b) we obtain the coefficient of determination $R_{i,j}^2$ which is interpreted as a normalized measure of how similar the two measurement series are (with $R^2 \to 1$ indicating perfect correlation). Based hereon, we construct a similarity matrix *S* that contains the calculated values for all pairs of IS, i.e. $S_{i,j} = R_{i,j}^2$. Prior to clustering, we obtain a rescaled similarity matrix *S'*, in which similarity values are linearly rescaled such that the lowest value is set to 0 and the largest value is set to 1.
We point out that the regression approach based on R^2 can be replaced by alternative methods for the pairwise evaluation of IS similarity, such as a Canberra distance[1].

Clustering of IS

Clustering algorithms are a suitable tool to identify sets of IS with correlated behaviors, which indicate a common clonal origin. Technically, each IS *i* within the rescaled similarity matrix *S'* is assigned to one cluster *c* out of a number of available clusters *k*. Clustering algorithms aim to maximize the inner-cluster similarities and minimize outer-cluster similarities to obtain an optimal assignment of the elements to the *k* clusters. As the number of expected clusters (i.e. clones, to which the IS are assigned) is unknown *a priori*, we apply the cluster algorithm multiple times (compare Figure 2d) while varying the number of clusters *k* between 2 and the number of IS retained after filtering minus 1 (the later representing the case that each except for one IS would belong to an individual clone; this bound was chosen as some clustering algorithms do not support assigning each element to its own cluster). We use *partitioning around medoids* (PAM) as a suitable clustering algorithm, which is a realization of the k-medoids algorithm[2]. In contrast to a *k-means* approach, PAM utilizes a given distance metric, while *k-means* intrinsically works on a Euclidean metric, which is not suitable in our case. Since the computational costs of PAM are not a limiting factor given the prior filtering step, we recommend using PAM for the given clustering problem, although the *MultIS* package also allows using other related clustering methods.

Evaluation of clusters

In order to compare clustering results for different numbers of clusters *k* we use the *silhouette score*[3]. This score quantifies the quality of a given clustering by comparing the inner-cluster distance with the outer-cluster distance.

Technically, a silhouette value *sil* of IS *i* is defined as:

$$sil(i) = \frac{oc(i) - ic(i)}{max\{ic(i), oc(i)\}}$$

Here *ic(i)* and *oc(i)* are the average distances of *i* to all IS within the same (inner) cluster and the IS within other (outer) clusters, respectively. We use the similarity matrix *S'* to obtain a corresponding distance matrix *S" = (1- S')*. The silhouette value of a cluster containing only a single IS is defined as *sil = 0*.

The overall silhouette score $\overline{sil}(k)$ for a given number of clusters *k* is defined as the average of all individual silhouette values over all IS (with n_{IS} referring to the total number of IS):

$$\overline{sil}(k) = \frac{\sum_{i=1}^{n_{IS}} sil(i)}{n_{IS}}$$

A silhouette score close to 1 indicates a higher inner-cluster similarity (i.e. lower distance) compared to a weaker outer-cluster similarity (i.e. larger distance). Calculating the corresponding silhouette scores *sil(k)* for different numbers of clusters $2 < k < n_{IS} - 1$, we consider the maximum *k*, named k^*, to identify the most likely number of clusters for the selected IS.

<u>Calculation of relative clonal abundances</u>

According to the optimal clustering, all IS are assigned to one of k^* clusters, each of which is interpreted as an independent clone. As a consequence, the clonal abundance $\widetilde{N_c}(t)$ is now given as the average over all IS abundances $\widehat{I_i}(t)$ assigned to the same cluster *c*. Additionally, the abundances of all IS that did not pass the filtering step (which will remain un-clustered to their source clones) have to be rescaled with an average number of IS per clone (representing an approximation of the average vector copy number VCN) to correct for their potential co-occurrence within the same clone. Although this approach does not allow to interpret minor IS as separate clones, it corrects their relative abundance to the observed clusters.

<u>Comparison of the reconstruction results with a known ground truth</u>

In order to evaluate the reconstruction quality, we compare the results of the clonal reconstruction process to the known ground truth that is available from simulated time courses and selected, annotated experiments. Technically, we calculate the adjusted Rand index (ARI) as a measure of overlap between the ground truth association and the clustering result. A value of ARI = 1 indicates a perfect clustering, i.e. IS that were associated to the same clone in the ground truth are also jointly recovered while other IS remain in distinct clusters. An ARI value of 0 would indicate a completely random assignment of IS to clusters. As the ARI only uses information about the co-occurrence of elements, it is label-independent.

Supplementary Note 2: Simulation model

The simulation model is adapted from Baldow et al.[4], in which time courses are generated from a stochastic, single cell-based model of clonal dynamics. The differentiation rate for each clone *c* is initialized from a normal distribution $\mathcal{N}(\mathrm{d}, \delta^2)$ and kept fixed thereafter. The standard deviation δ of this distribution is the model parameter which we use to tune the extent of the clonal differences and influence how fast the system will converge towards clonal dominance. The proliferation rate is identical for all clones and regulated by a logistic growth function with overall carrying capacity *K* and maximal proliferation rate p_{max}. Over time, the competing clones increase or decrease in size. For each clone, the number of unique clonal IS is determined by sampling from a Poisson distribution with mean λ, which reflects the average vector copy number. All IS *i* from the same clone change their abundance $I_i(t)$ along with the abundance of the clone from which they originate.

The model simulations are further extended to reflect changing clonal contributions to different hematological lineages. Technically, we introduced an artificial clonal shift such that prior to each measurement the size of a particular clone is multiplied by a random number drawn from a normal distribution with parameters $\mathcal{N}(1, \nu^2)$ thereby affecting the abundance of all IS for each particular clone. For $\nu = 0$, the final clone sizes correspond to the clonal time courses (i.e. $I_i^*(t) = I_i(t)$), while for values of $\nu > 0$ clone sizes for each measurement point are randomly and moderately varied.

The chosen model parameters are provided in Supplementary Table S4.

Supplementary Note 3: Experimental data

Validation assay.
Chronic Myelogenous Leukemia (K562) cell clones were transduced with a lentiviral vector expressing GFP under the control of the Spleen Focus Forming Virus promoter[5], using a multiplicity of infection (MOI) of 10. Two weeks after transduction, single cell clones were isolated from the GFP+ population of cells by FACS sorting and cultured in IMDM-medium for several weeks. For each clone, the LV vector/genome junctions were retrieved by sonication-based linker-mediated (SLiM) PCR. In Supplementary Table S2 we reported the different IS identified in each of the four clones used in the validation assay. Additionally, human B-lymphoblastoid cells (JY cells) were transduced in bulk with a lentiviral vector expressing GFP under the control of PGK promoter[6] and using a multiplicity of infection of 1. The VCN in the K562 cell clones and JY was determined by ddPCR.

Clonal tracing in mice.
Lin- cells were collected from n=12 Cdkn2a$^{-/-}$ donor mice, half female and half male, aged eight weeks. Transduced cells were transplanted in eight female, lethally irradiated wild-type C57BL6/J mice, aged 8 weeks. All mice were bred and kept in a dedicated pathogen-free animal facility where 12-hour light/12-hour dark cycle is used, and the colony room was maintained at 22°C±2 C with humidity of 55±5%. In agreement with previously published data[6], mice receiving Cdkn2a$^{-/-}$ Lin- cells transduced with SINLV.PGK.GFP developed tumors similarly to the mock-control group. Mice were euthanized when they showed signs of severe sickness.
To recover enough starting material for the sorting procedure, DNA extraction and subsequent molecular analyses, an equal amount of blood from a cohort of two to three different animals was pooled together prior to sorting. The composition of each pool was maintained constant throughout the whole experiment, so that each pool is composed by the same mice over time. Tissue samples taken at euthanasia (bone marrow, spleen, thymus, and lymph nodes) were analyzed separately for each animal.

Retrieval of IS from cell DNA / SLiM PCR
For the retrieval of vector IS from genomic DNA, we adopted a SLiM-PCR method similar to the previously described[7]. This procedure has been developed to overcome the limitations of the previously used method, namely linear amplification mediated (LAM)-PCR[8]. Briefly, for the applied SLiM-PCR procedure genomic DNA was sheared using a Covaris E220 Ultrasonicator (Covaris Inc., Woburn, MA.), and split in three technical replicates. The fragmented DNA was then subjected to end repair and 3' adenylation using the NEBNext® Ultra™ DNA Library Prep Kit for Illumina® (New England Biolabs, Ipswich, MA.), and then ligated (DNA Technologies ligation kit, Skokie, IL.) to the linker cassettes (LC) containing a sequence barcode for sample identification and all the sequences required for the Read 2 Illumina paired end sequencing. Ligation products were then subjected to 35 cycles of exponential PCR and next ten additional PCR cycles were done to add sequences required for sequencing. Finally, the amplification products were sequenced using the Illumina NextSeq 550 sequencing platform (Illumina, San Diego, CA.). A more detailed technical description of the Slim-PCR approach was recently reported in the supplementary materials of Cesana et al.[9].

Sequencing reads were then processed by a dedicated bioinformatics pipeline (VISPA2) as described before[10]. Briefly, paired sequence reads are filtered for quality standards, barcodes identified for sample de-multiplexing of the sequence reads, the cellular genomic sequence mapped on the reference Human genome (Human Genome_GRCh37/hg19 Feb. 2019) or mouse (Mouse Genome_mm9) and the nearest RefSeq gene assigned to each unambiguously mapped integration site. For the quantification of the abundance of each clone we adopted an estimation method previously described[11] where the abundance is determined

by the number of different DNA fragments containing the same vector/cell genome junctions flanked by a genomic segment variable in size depending on the shear site position, unique for each different cell genome present in the starting cell population. Therefore, the number of different shear sites assigned to an IS will be proportional to the initial number of contributing cells, allowing to estimate the clonal abundance in the starting sample avoiding the biases introduced by PCR amplification.

Although the efficiency of SLiM-PCR depends on the amount of reads per sequencing pool and the amount of DNA analyzed, usually SLiM-PCR sequencing libraries are assembled with equimolar amounts of DNA for each PCR reaction. This strategy reduces artifacts on library composition and avoids compromising the sequence content of the pool. For this reason, PCR reactions will end up with a similar number of reads (on average between 1 and 10 million reads, depending on the sequencing platform and the number of samples per pool) thus limiting the impact of any biases in IS abundance. Nevertheless, once we identified IS from the NGS library (i.e. Illumina sequencing pool) using VISPA2, our downstream analytical pipeline removes PCR reactions with too few reads (>3 standard deviations from the average). All our data presented here has been processed under these conditions.

Droplet digital (dd)PCR

The abundance of each K562 clone in the DNA mixes was measured by ddPCR using the QuantaLife ddPCR system. Custom-made ddPCR assays were designed to specifically amplify at least one of the LV vector genome junctions retrieved in each clone. Human GAPDH ddPCR assay was used as the housekeeping gene. 20-30 nanograms of genomic DNA for each mix were used for PCR amplification performed in triplicate and in a final volume of 20 μl. Plates were quantified in a QuantaLife droplet reader, and the concentrations of the targets in the samples were determined using QuantaSoft software (Version 1.7.4.0917).

IS measurements from a study on rhesus macaques

We retrieved publicly available data from a study on rhesus macaques receiving autologous transplantation with HSPCs that were transduced with a lentiviral vector[12]. One of the animals developed abnormal clonal hematopoiesis affecting different blood lineages, for which full time course data is available. Technically, genomic barcodes were used to quantify the abundance of individual IS in myeloid and lymphatic compartments at different time points. It could be experimentally verified that the six barcodes accounting for up to 100% of the observed barcodes in some of the lineages derive from the same clone. For this clone, three further IS could be confirmed using redesigned primers, which are not present in the time course data. The analysis of this data using *MultIS* explicitly excludes the time course data on nucleated red blood cells, as this compartment is solely composed of cells from the dominating clone while such data is generally not available in a respective GT data set.

Supplementary Note 4: R package *MultIS*

The R package *MultIS* provides an implementation of the functionality presented in the accompanying manuscript. The package is available from the Comprehensive R Archive Network (CRAN) under the Lesser GNU Public License Version 3 (LGPLv3).

The package comprises the basic methods used for filtering, calculation of similarities, clustering, and the evaluation of clusters. Furthermore, it provides corresponding visualization routines for each step in the pipeline. Along with the package we provide a vignette illustrating an exemplary workflow.

Methods for normalizing and filtering IS read data

MultIS implements functionality for the normalization and filtering of time course data with an intuitive naming of the respective functions.

As a starting point, the application of our R package *MultIS* requires a simple matrix with unique identifiers for the IS in the rownames and the corresponding read counts for each available measurement in the columns.

The first recommended step to achieve comparability within one experimental series is a normalization of IS counts to a relative scale for each measurement ("MultIS::convert_columnwise_relative"). For the subsequent filtering step, different methods can be adapted, e.g. with respect to an absolute number of selected, most abundant IS (e.g. using the n largest IS only: "MultIS::filter_at_tp_biggest_n") or with respect to a threshold value (e.g. I^{*rel} >1% at the final time point or in given tissue samples, see "MultIS::filter_at_tp_min"). The filtering methods also include matching for patterns in measurements ("MultIS::filter_match") or in IS names. *MultIS* further includes different functions to transform measurement names ("MultIS::filter_measurement_names") and shorten IS identifiers to their shortest distinct prefix ("MultIS::filter_is_names").

Measuring similarities between IS

To measure the similarity between IS we construct a similarity matrix *S* that contains each pairwise rating of similarity ("MultIS::get_similarity_matrix"). We recommend using the R^2 metric (coefficient of determination obtained from a linear regression through the origin), while other metrics can be considered depending on the available data set. Technically, *MultIS* additionally supports all other methods provided by the "dist" function the "stats" package in *R* such as the Euclidean, Manhattan, or Canberra distance[1] via the "method" argument of the "get_similarity_matrix" function.

Clustering IS

We use partitioning around medoids (PAM)[2] as a suitable method to identify clusters of IS with correlated behaviors ("MultIS::reconstruct"). However, the cluster function can be called with other "method" arguments than "kmedoids" (with its implementation PAM), such as any method implemented in "stats::hclust".

Rating and finding the optimal number of clusters

In the experimental setting the number of clones in a sample is unknown. Thus, our method aims towards estimating this number from the correlation structure within the rescaled similarity matrix *S'*. Supervised clustering methods require a predefined number of expected clusters *k*. We repeatedly apply the clustering for all values of *k* between 2 and the number of IS – 1 ("MultIS::find_best_nr_cluster"). We suggest to use the silhouette score[3] to compare the cluster results for different given values of *k* and to identify the optimal number *k** that maximizes the inner and minimizes the outer-cluster similarity. Alternative methods can be used instead, e.g. the SD index[13], the point-biserial index[14-16], or the Dunn index[17]. Using the best number of clusters, the user can reconstruct the clonal assignment for the IS retained after filtering. Based on this assignment, a corrected "clonal abundance" can be calculated ("MultIS::normalize_timecourse").

Methods for data visualization

Each step in the reconstruction pipeline of *MultIS* returns an object that can directly be used with R's "plot" function to visualize the results and to give the user a quick overview of the data. The "plot" function uses the dispatch mechanism of R to automatically call an appropriate plotting function from the *MultIS* package.

Time courses
Time courses are visualized using a stacked area plot that shows their relative contribution over time. The measurements can either be time points or combinations of time points and cell types. If the cell type is encoded in the measurement name, we provide the functionality to extract the cell type part from the naming convention of the measurement and use this as facets of different sub-plots. Coloring schemes can be applied to ensure an internal consistency across different plots.

Similarity matrices
Similarity matrices *S* can be efficiently visualized as heatmaps. Internally, the heat map function also performs a hierarchical clustering to display similar IS adjacent to each other.

Clustered IS and similarities
We recommend a spring model to visualize the results of different clustering methods. IS are laid out as nodes on a plane and connected via springs. The strength of these springs is proportional to the similarity contained in the similarity matrix *S*. Thus, more closely related IS take a position that is closer to each other in this plot. The shading of the edges refers to the mutual similarity of any two IS. The coloring of the nodes (i.e. IS) indicates whether they are associated with the same cluster. If an additional ground truth is known, an inner and outer coloring of the nodes can be used to distinguish the reconstructed and the known associations.

Supplementary Note 5: Data provision and reproducibility

We provide a collection of R-script files along with all the necessary data sets to fully reproduce all steps within our manuscript (https://gitlab.com/imb-dev/clonal-reconstruction-figures). The scripts rely upon the functionality of the *MultIS* package, available at CRAN and outlined above. While the script files are included for reasons of reproducibility rather than to serve as a tutorial, we refer the interested user to the vignette included in the *MultIS* package.

The macaque data set[12] can be obtained from the GEO database, accession number GSE153130 at https://www.ncbi.nlm.nih.gov/geo/download/?acc=GSE153130, file GSE153130_zl34_barcode_count_matrix.txt.gz

Supplementary Note 6: Reagents, PCR Primers and barcoded linker cassettes adopted for SLiM-PCR protocols

Instruments and Reagents

Instrument	Company
Qubit 3.0 Fluorometer	ThermoFisher Scientific
E220 Focused-ultrasonicator	Covaris
MyCycler	Biorad
LabChip GX Touch HT	PerkinElmer
Microlab Star	Hamilton
Microlab Starlet	Hamilton
Viia7	ThermoFisher Scientific

General supply	ID	Company
Conductive 50 ul Filter Tips in Frames	235948	Hamilton
Conductive 300 ul Filter Tips in Frames	235903	Hamilton
Conductive 1000 ul Filter Tips in Frames	235905	Hamilton
8 microTUBE Strip AFA Fiber	520053	Covaris
Rack 12 place 8 microTUBE Strip	500191	Covaris
Twin.tec PCR Plate 96, semi-skirted, colorless	951020303	Eppendorf
Hard-Shell PCR Plates, 384-Well CLR-WHT	HSP3805	Biorad
Qubit® Assay Tubes	Q32856	ThermoFisher Scientific

Reagents	ID	Company
Qubit® dsDNA HS Assay Kit 500 assays	Q32854	ThermoFisher Scientific
DNA Clean & Concentrator Kit	D4013	Zymo Research
NEBNext Ultra DNA Library Prep Kit for Illumina	E7370L	New England BioLabs
Agencourt AMPureXP-PCR Purification	A63881	Beckman Coluter
Ethanol	2860	Fluka Analytical
Trizma hydrochloride buffer solution pH=8	T2694	Sigma
Water	W3500	Sigma
dNTPS 10 nM	R0186	Fermentas
PCR Buffer 10X	1005479	Qiagen
Taq DNA Polymerase	1005476	Qiagen
Illumina Library Quantification Kit Universal qPCR Mix	KK4824	Kapa Biosystems
Free Adapter Blocking Reagent	20024145	Illumina
dATP 100 mM	Thermo Scientific	#R0141
dTTP 100 mM	Thermo Scientific	#R0172
dGTP 100 mM	Thermo Scientific	#R0162
dCTP 100 mM	Thermo Scientific	#R0152
PCR Buffer, 10x with 15 mM $MgCl_2$	Quiagen	1005479

PCR Primers and Linker cassettes sequences

All PCR primers were synthetized at Integrated DNA Technologies (Coralville, Iowa).

Barcoded Linker cassettes

Linker cassettes are generated by annealing a long and short oligo and delivered as ready-to-use 15 micromolar. The long oligo contains the 8-nucleotide barcode for sample identification and the 12-nucleotide random sequence used for clonal size estimation.

Name	Sequence	Sequence 2	Barcode
LC1	GACGTGTGCTCTTCCGATCTNNNNNNNNNNNNGAACGGTTGTCACCGTGTCGTCAATCC*T(25252525)	/5Phos/GGATTGACGACACGGTGAC/3C6/	GAACGGTT
LC2	GACGTGTGCTCTTCCGATCTNNNNNNNNNNNNAACAACCAGTCACCGTGTCGTCAATCC*T(25252525)	/5Phos/GGATTGACGACACGGTGAC/3C6/	AACAACCA
LC3	GACGTGTGCTCTTCCGATCTNNNNNNNNNNNNTGCTTGCTGTCACCGTGTCGTCAATCC*T(25252525)	/5Phos/GGATTGACGACACGGTGAC/3C6/	TGCTTGCT
LC4	GACGTGTGCTCTTCCGATCTNNNNNNNNNNNNAACGCTTAGTCACCGTGTCGTCAATCC*T(25252525)	/5Phos/GGATTGACGACACGGTGAC/3C6/	AACGCTTA
LC5	GACGTGTGCTCTTCCGATCTNNNNNNNNNNNNCAAGTCGTGTCACCGTGTCGTCAATCC*T(25252525)	/5Phos/GGATTGACGACACGGTGAC/3C6/	CAAGTCGT
LC6	GACGTGTGCTCTTCCGATCTNNNNNNNNNNNNAACTCACCGTCACCGTGTCGTCAATCC*T(25252525)	/5Phos/GGATTGACGACACGGTGAC/3C6/	AACTCACC
LC7	GACGTGTGCTCTTCCGATCTNNNNNNNNNNNNCCTGTCAAGTCACCGTGTCGTCAATCC*T(25252525)	/5Phos/GGATTGACGACACGGTGAC/3C6/	CCTGTCAA
LC8	GACGTGTGCTCTTCCGATCTNNNNNNNNNNNNAAGAGATCGTCACCGTGTCGTCAATCC*T(25252525)	/5Phos/GGATTGACGACACGGTGAC/3C6/	AAGAGATC
LC9	GACGTGTGCTCTTCCGATCTNNNNNNNNNNNNCGTCCATTGTCACCGTGTCGTCAATCC*T(25252525)	/5Phos/GGATTGACGACACGGTGAC/3C6/	CGTCCATT
LC10	GACGTGTGCTCTTCCGATCTNNNNNNNNNNNNAAGGTACAGTCACCGTGTCGTCAATCC*T(25252525)	/5Phos/GGATTGACGACACGGTGAC/3C6/	AAGGTACA
LC11	GACGTGTGCTCTTCCGATCTNNNNNNNNNNNNAGCTTCAGGTCACCGTGTCGTCAATCC*T(25252525)	/5Phos/GGATTGACGACACGGTGAC/3C6/	AGCTTCAG
LC12	GACGTGTGCTCTTCCGATCTNNNNNNNNNNNNAATGTTGCGTCACCGTGTCGTCAATCC*T(25252525)	/5Phos/GGATTGACGACACGGTGAC/3C6/	AATGTTGC
LC13	GACGTGTGCTCTTCCGATCTNNNNNNNNNNNNCATTGACGGTCACCGTGTCGTCAATCC*T(25252525)	/5Phos/GGATTGACGACACGGTGAC/3C6/	CATTGACG
LC14	GACGTGTGCTCTTCCGATCTNNNNNNNNNNNNACACAGAAGTCACCGTGTCGTCAATCC*T(25252525)	/5Phos/GGATTGACGACACGGTGAC/3C6/	ACACAGAA
LC15	GACGTGTGCTCTTCCGATCTNNNNNNNNNNNNCCTTCCATGTCACCGTGTCGTCAATCC*T(25252525)	/5Phos/GGATTGACGACACGGTGAC/3C6/	CCTTCCAT
LC16	GACGTGTGCTCTTCCGATCTNNNNNNNNNNNNACAGATTCGTCACCGTGTCGTCAATCC*T(25252525)	/5Phos/GGATTGACGACACGGTGAC/3C6/	ACAGATTC
LC17	GACGTGTGCTCTTCCGATCTNNNNNNNNNNNNCTTCGGTTGTCACCGTGTCGTCAATCC*T(25252525)	/5Phos/GGATTGACGACACGGTGAC/3C6/	CTTCGGTT
LC18	GACGTGTGCTCTTCCGATCTNNNNNNNNNNNNACATTGGCGTCACCGTGTCGTCAATCC*T(25252525)	/5Phos/GGATTGACGACACGGTGAC/3C6/	ACATTGGC
LC19	GACGTGTGCTCTTCCGATCTNNNNNNNNNNNNGAACCTTCGTCACCGTGTCGTCAATCC*T(25252525)	/5Phos/GGATTGACGACACGGTGAC/3C6/	GAACCTTC
LC20	GACGTGTGCTCTTCCGATCTNNNNNNNNNNNNACCTCCAAGTCACCGTGTCGTCAATCC*T(25252525)	/5Phos/GGATTGACGACACGGTGAC/3C6/	ACCTCCAA
LC21	GACGTGTGCTCTTCCGATCTNNNNNNNNNNNNCTCCAATCGTCACCGTGTCGTCAATCC*T(25252525)	/5Phos/GGATTGACGACACGGTGAC/3C6/	CTCCAATC
LC22	GACGTGTGCTCTTCCGATCTNNNNNNNNNNNNACGTATCAGTCACCGTGTCGTCAATCC*T(25252525)	/5Phos/GGATTGACGACACGGTGAC/3C6/	ACGTATCA
LC23	GACGTGTGCTCTTCCGATCTNNNNNNNNNNNNGTTGGCATGTCACCGTGTCGTCAATCC*T(25252525)	/5Phos/GGATTGACGACACGGTGAC/3C6/	GTTGGCAT
LC24	GACGTGTGCTCTTCCGATCTNNNNNNNNNNNNAGAGTCAAGTCACCGTGTCGTCAATCC*T(25252525)	/5Phos/GGATTGACGACACGGTGAC/3C6/	AGAGTCAA
LC25	GACGTGTGCTCTTCCGATCTNNNNNNNNNNNNCGCGTATTGTCACCGTGTCGTCAATCC*T(25252525)	/5Phos/GGATTGACGACACGGTGAC/3C6/	CGCGTATT
LC26	GACGTGTGCTCTTCCGATCTNNNNNNNNNNNNAGATGTACGTCACCGTGTCGTCAATCC*T(25252525)	/5Phos/GGATTGACGACACGGTGAC/3C6/	AGATGTAC
LC27	GACGTGTGCTCTTCCGATCTNNNNNNNNNNNNCACTGTAGGTCACCGTGTCGTCAATCC*T(25252525)	/5Phos/GGATTGACGACACGGTGAC/3C6/	CACTGTAG
LC28	GACGTGTGCTCTTCCGATCTNNNNNNNNNNNNAGCAGGAAGTCACCGTGTCGTCAATCC*T(25252525)	/5Phos/GGATTGACGACACGGTGAC/3C6/	AGCAGGAA

Name	Sequence	Sequence 2	Barcode
LC29	GACGTGTGCTCTTCCGATCTNNNNNNNNNNNNTCAGACACGTCACC<br>GTGTCGTCAATCC*T(25252525)	/5Phos/GGATTGACGACACGGTGAC/3C6<br>/	TCAGACA<br>C
LC30	GACGTGTGCTCTTCCGATCTNNNNNNNNNNNNAGGCTAACGTCACC<br>GTGTCGTCAATCC*T(25252525)	/5Phos/GGATTGACGACACGGTGAC/3C6<br>/	AGGCTAA<br>C
LC31	GACGTGTGCTCTTCCGATCTNNNNNNNNNNNNTGCGTAACGTCACC<br>GTGTCGTCAATCC*T(25252525)	/5Phos/GGATTGACGACACGGTGAC/3C6<br>/	TGCGTAA<br>C
LC32	GACGTGTGCTCTTCCGATCTNNNNNNNNNNNNAGTCACTAGTCACC<br>GTGTCGTCAATCC*T(25252525)	/5Phos/GGATTGACGACACGGTGAC/3C6<br>/	AGTCACT<br>A
LC33	GACGTGTGCTCTTCCGATCTNNNNNNNNNNNNCCAACGAAGTCACC<br>GTGTCGTCAATCC*T(25252525)	/5Phos/GGATTGACGACACGGTGAC/3C6<br>/	CCAACGA<br>A
LC34	GACGTGTGCTCTTCCGATCTNNNNNNNNNNNNATAGCGACGTCACC<br>GTGTCGTCAATCC*T(25252525)	/5Phos/GGATTGACGACACGGTGAC/3C6<br>/	ATAGCGA<br>C
LC35	GACGTGTGCTCTTCCGATCTNNNNNNNNNNNNTCGATGACGTCACC<br>GTGTCGTCAATCC*T(25252525)	/5Phos/GGATTGACGACACGGTGAC/3C6<br>/	TCGATGA<br>C
LC36	GACGTGTGCTCTTCCGATCTNNNNNNNNNNNNATCCTGTAGTCACC<br>GTGTCGTCAATCC*T(25252525)	/5Phos/GGATTGACGACACGGTGAC/3C6<br>/	ATCCTGTA
LC37	GACGTGTGCTCTTCCGATCTNNNNNNNNNNNNCTCTTGTCGTCACC<br>GTGTCGTCAATCC*T(25252525)	/5Phos/GGATTGACGACACGGTGAC/3C6<br>/	CTCTTGTC
LC38	GACGTGTGCTCTTCCGATCTNNNNNNNNNNNNATTGAGGAGTCACC<br>GTGTCGTCAATCC*T(25252525)	/5Phos/GGATTGACGACACGGTGAC/3C6<br>/	ATTGAGG<br>A
LC39	GACGTGTGCTCTTCCGATCTNNNNNNNNNNNNGATCTCAGGTCACC<br>GTGTCGTCAATCC*T(25252525)	/5Phos/GGATTGACGACACGGTGAC/3C6<br>/	GATCTCA<br>G
LC40	GACGTGTGCTCTTCCGATCTNNNNNNNNNNNNCAACCACAGTCACC<br>GTGTCGTCAATCC*T(25252525)	/5Phos/GGATTGACGACACGGTGAC/3C6<br>/	CAACCAC<br>A
LC41	GACGTGTGCTCTTCCGATCTNNNNNNNNNNNNCGATCGATGTCACC<br>GTGTCGTCAATCC*T(25252525)	/5Phos/GGATTGACGACACGGTGAC/3C6<br>/	CGATCGA<br>T
LC42	GACGTGTGCTCTTCCGATCTNNNNNNNNNNNNCAAGGAGCGTCAC<br>CGTGTCGTCAATCC*T(25252525)	/5Phos/GGATTGACGACACGGTGAC/3C6<br>/	CAAGGAG<br>C
LC43	GACGTGTGCTCTTCCGATCTNNNNNNNNNNNNTACTCCAGGTCACC<br>GTGTCGTCAATCC*T(25252525)	/5Phos/GGATTGACGACACGGTGAC/3C6<br>/	TACTCCAG
LC44	GACGTGTGCTCTTCCGATCTNNNNNNNNNNNNCACCTTACGTCACC<br>GTGTCGTCAATCC*T(25252525)	/5Phos/GGATTGACGACACGGTGAC/3C6<br>/	CACCTTAC
LC45	GACGTGTGCTCTTCCGATCTNNNNNNNNNNNNTACGACGTGTCACC<br>GTGTCGTCAATCC*T(25252525)	/5Phos/GGATTGACGACACGGTGAC/3C6<br>/	TACGACG<br>T
LC46	GACGTGTGCTCTTCCGATCTNNNNNNNNNNNNCAGATCTGGTCACC<br>GTGTCGTCAATCC*T(25252525)	/5Phos/GGATTGACGACACGGTGAC/3C6<br>/	CAGATCT<br>G
LC47	GACGTGTGCTCTTCCGATCTNNNNNNNNNNNNACCGAATGGTCACC<br>GTGTCGTCAATCC*T(25252525)	/5Phos/GGATTGACGACACGGTGAC/3C6<br>/	ACCGAAT<br>G
LC48	GACGTGTGCTCTTCCGATCTNNNNNNNNNNNNCATACCAAGTCACC<br>GTGTCGTCAATCC*T(25252525)	/5Phos/GGATTGACGACACGGTGAC/3C6<br>/	CATACCA<br>A
LC49	GACGTGTGCTCTTCCGATCTNNNNNNNNNNNNCCAACACTGTCACC<br>GTGTCGTCAATCC*T(25252525)	/5Phos/GGATTGACGACACGGTGAC/3C6<br>/	CCAACACT
LC50	GACGTGTGCTCTTCCGATCTNNNNNNNNNNNNCCAGTTCAGTCACC<br>GTGTCGTCAATCC*T(25252525)	/5Phos/GGATTGACGACACGGTGAC/3C6<br>/	CCAGTTCA
LC51	GACGTGTGCTCTTCCGATCTNNNNNNNNNNNNAATTCCGGGTCACC<br>GTGTCGTCAATCC*T(25252525)	/5Phos/GGATTGACGACACGGTGAC/3C6<br>/	AATTCCG<br>G
LC52	GACGTGTGCTCTTCCGATCTNNNNNNNNNNNNCCGAAGTAGTCACC<br>GTGTCGTCAATCC*T(25252525)	/5Phos/GGATTGACGACACGGTGAC/3C6<br>/	CCGAAGT<br>A
LC53	GACGTGTGCTCTTCCGATCTNNNNNNNNNNNNACTCAACGGTCACC<br>GTGTCGTCAATCC*T(25252525)	/5Phos/GGATTGACGACACGGTGAC/3C6<br>/	ACTCAAC<br>G
LC54	GACGTGTGCTCTTCCGATCTNNNNNNNNNNNNCCGTGAGAGTCACC<br>GTGTCGTCAATCC*T(25252525)	/5Phos/GGATTGACGACACGGTGAC/3C6<br>/	CCGTGAG<br>A
LC55	GACGTGTGCTCTTCCGATCTNNNNNNNNNNNNATCTCCTGGTCACC<br>GTGTCGTCAATCC*T(25252525)	/5Phos/GGATTGACGACACGGTGAC/3C6<br>/	ATCTCCTG
LC56	GACGTGTGCTCTTCCGATCTNNNNNNNNNNNNCCTCCTGAGTCACC<br>GTGTCGTCAATCC*T(25252525)	/5Phos/GGATTGACGACACGGTGAC/3C6<br>/	CCTCCTGA
LC57	GACGTGTGCTCTTCCGATCTNNNNNNNNNNNNGCCTTAACGTCACC<br>GTGTCGTCAATCC*T(25252525)	/5Phos/GGATTGACGACACGGTGAC/3C6<br>/	GCCTTAAC
LC58	GACGTGTGCTCTTCCGATCTNNNNNNNNNNNNCGAACTTAGTCACC<br>GTGTCGTCAATCC*T(25252525)	/5Phos/GGATTGACGACACGGTGAC/3C6<br>/	CGAACTT<br>A
LC59	GACGTGTGCTCTTCCGATCTNNNNNNNNNNNNGTGAGACTGTCACC<br>GTGTCGTCAATCC*T(25252525)	/5Phos/GGATTGACGACACGGTGAC/3C6<br>/	GTGAGAC<br>T
LC60	GACGTGTGCTCTTCCGATCTNNNNNNNNNNNNCGACTGGAGTCACC<br>GTGTCGTCAATCC*T(25252525)	/5Phos/GGATTGACGACACGGTGAC/3C6<br>/	CGACTGG<br>A
LC61	GACGTGTGCTCTTCCGATCTNNNNNNNNNNNNGAGTAGAGGTCAC<br>CGTGTCGTCAATCC*T(25252525)	/5Phos/GGATTGACGACACGGTGAC/3C6<br>/	GAGTAGA<br>G
LC62	GACGTGTGCTCTTCCGATCTNNNNNNNNNNNNCGCTGATCGTCACC<br>GTGTCGTCAATCC*T(25252525)	/5Phos/GGATTGACGACACGGTGAC/3C6<br>/	CGCTGAT<br>C

Name	Sequence	Sequence 2	Barcode
LC63	GACGTGTGCTCTTCCGATCTNNNNNNNNNNNNNNCTCGACTTGTCACC<br>GTGTCGTCAATCC*T(25252525)	/5Phos/GGATTGACGACACGGTGAC/3C6<br>/	CTCGACTT
LC64	GACGTGTGCTCTTCCGATCTNNNNNNNNNNNNNNCTAAGGTCGTCACC<br>GTGTCGTCAATCC*T(25252525)	/5Phos/GGATTGACGACACGGTGAC/3C6<br>/	CTAAGGT<br>C
LC65	GACGTGTGCTCTTCCGATCTNNNNNNNNNNNNNNGTCGATTGGTCACC<br>GTGTCGTCAATCC*T(25252525)	/5Phos/GGATTGACGACACGGTGAC/3C6<br>/	GTCGATT<br>G
LC66	GACGTGTGCTCTTCCGATCTNNNNNNNNNNNNNNCTGAGCCAGTCACC<br>GTGTCGTCAATCC*T(25252525)	/5Phos/GGATTGACGACACGGTGAC/3C6<br>/	CTGAGCC<br>A
LC67	GACGTGTGCTCTTCCGATCTNNNNNNNNNNNNNNCTCGAACAGTCACC<br>GTGTCGTCAATCC*T(25252525)	/5Phos/GGATTGACGACACGGTGAC/3C6<br>/	CTCGAAC<br>A
LC68	GACGTGTGCTCTTCCGATCTNNNNNNNNNNNNNNCTGTAGCCGTCACC<br>GTGTCGTCAATCC*T(25252525)	/5Phos/GGATTGACGACACGGTGAC/3C6<br>/	CTGTAGC<br>C
LC69	GACGTGTGCTCTTCCGATCTNNNNNNNNNNNNNNGTAGCGTAGTCACC<br>GTGTCGTCAATCC*T(25252525)	/5Phos/GGATTGACGACACGGTGAC/3C6<br>/	GTAGCGT<br>A
LC70	GACGTGTGCTCTTCCGATCTNNNNNNNNNNNNNNGAATCTGAGTCACC<br>GTGTCGTCAATCC*T(25252525)	/5Phos/GGATTGACGACACGGTGAC/3C6<br>/	GAATCTG<br>A
LC71	GACGTGTGCTCTTCCGATCTNNNNNNNNNNNNNNATTCCGCTGTCACC<br>GTGTCGTCAATCC*T(25252525)	/5Phos/GGATTGACGACACGGTGAC/3C6<br>/	ATTCCGCT
LC72	GACGTGTGCTCTTCCGATCTNNNNNNNNNNNNNNGACTAGTAGTCACC<br>GTGTCGTCAATCC*T(25252525)	/5Phos/GGATTGACGACACGGTGAC/3C6<br>/	GACTAGT<br>A
LC73	GACGTGTGCTCTTCCGATCTNNNNNNNNNNNNNNCTATGCCTGTCACC<br>GTGTCGTCAATCC*T(25252525)	/5Phos/GGATTGACGACACGGTGAC/3C6<br>/	CTATGCCT
LC74	GACGTGTGCTCTTCCGATCTNNNNNNNNNNNNNNGAGTTAGCGTCACC<br>GTGTCGTCAATCC*T(25252525)	/5Phos/GGATTGACGACACGGTGAC/3C6<br>/	GAGTTAG<br>C
LC75	GACGTGTGCTCTTCCGATCTNNNNNNNNNNNNNNTCGAGAGTGTCACC<br>GTGTCGTCAATCC*T(25252525)	/5Phos/GGATTGACGACACGGTGAC/3C6<br>/	TCGAGAG<br>T
LC76	GACGTGTGCTCTTCCGATCTNNNNNNNNNNNNNNGATGAATCGTCACC<br>GTGTCGTCAATCC*T(25252525)	/5Phos/GGATTGACGACACGGTGAC/3C6<br>/	GATGAAT<br>C
LC77	GACGTGTGCTCTTCCGATCTNNNNNNNNNNNNNNGATGGAGTGTCAC<br>CGTGTCGTCAATCC*T(25252525)	/5Phos/GGATTGACGACACGGTGAC/3C6<br>/	GATGGAG<br>T
LC78	GACGTGTGCTCTTCCGATCTNNNNNNNNNNNNNNGCCACATAGTCACC<br>GTGTCGTCAATCC*T(25252525)	/5Phos/GGATTGACGACACGGTGAC/3C6<br>/	GCCACAT<br>A
LC79	GACGTGTGCTCTTCCGATCTNNNNNNNNNNNNNNGTACGATCGTCACC<br>GTGTCGTCAATCC*T(25252525)	/5Phos/GGATTGACGACACGGTGAC/3C6<br>/	GTACGAT<br>C
LC80	GACGTGTGCTCTTCCGATCTNNNNNNNNNNNNNNGCTAACGAGTCACC<br>GTGTCGTCAATCC*T(25252525)	/5Phos/GGATTGACGACACGGTGAC/3C6<br>/	GCTAACG<br>A
LC81	GACGTGTGCTCTTCCGATCTNNNNNNNNNNNNNNTACCGGATGTCACC<br>GTGTCGTCAATCC*T(25252525)	/5Phos/GGATTGACGACACGGTGAC/3C6<br>/	TACCGGA<br>T
LC82	GACGTGTGCTCTTCCGATCTNNNNNNNNNNNNNNGGAGAACAGTCAC<br>CGTGTCGTCAATCC*T(25252525)	/5Phos/GGATTGACGACACGGTGAC/3C6<br>/	GGAGAAC<br>A
LC83	GACGTGTGCTCTTCCGATCTNNNNNNNNNNNNNNAACACGCTGTCACC<br>GTGTCGTCAATCC*T(25252525)	/5Phos/GGATTGACGACACGGTGAC/3C6<br>/	AACACGC<br>T
LC84	GACGTGTGCTCTTCCGATCTNNNNNNNNNNNNNNGTACGCAAGTCACC<br>GTGTCGTCAATCC*T(25252525)	/5Phos/GGATTGACGACACGGTGAC/3C6<br>/	GTACGCA<br>A
LC85	GACGTGTGCTCTTCCGATCTNNNNNNNNNNNNNNTTCCTCCTGTCACC<br>GTGTCGTCAATCC*T(25252525)	/5Phos/GGATTGACGACACGGTGAC/3C6<br>/	TTCCTCCT
LC86	GACGTGTGCTCTTCCGATCTNNNNNNNNNNNNNNGTCTGTCAGTCACC<br>GTGTCGTCAATCC*T(25252525)	/5Phos/GGATTGACGACACGGTGAC/3C6<br>/	GTCTGTCA
LC87	GACGTGTGCTCTTCCGATCTNNNNNNNNNNNNNNGAGAAGGTGTCAC<br>CGTGTCGTCAATCC*T(25252525)	/5Phos/GGATTGACGACACGGTGAC/3C6<br>/	GAGAAGG<br>T
LC88	GACGTGTGCTCTTCCGATCTNNNNNNNNNNNNNNTAGGATGAGTCACC<br>GTGTCGTCAATCC*T(25252525)	/5Phos/GGATTGACGACACGGTGAC/3C6<br>/	TAGGATG<br>A
LC89	GACGTGTGCTCTTCCGATCTNNNNNNNNNNNNNNTGATAGGCGTCACC<br>GTGTCGTCAATCC*T(25252525)	/5Phos/GGATTGACGACACGGTGAC/3C6<br>/	TGATAGG<br>C
LC90	GACGTGTGCTCTTCCGATCTNNNNNNNNNNNNNNTCCGTCTAGTCACC<br>GTGTCGTCAATCC*T(25252525)	/5Phos/GGATTGACGACACGGTGAC/3C6<br>/	TCCGTCTA
LC91	GACGTGTGCTCTTCCGATCTNNNNNNNNNNNNNNAGATACGGGTCAC<br>CGTGTCGTCAATCC*T(25252525)	/5Phos/GGATTGACGACACGGTGAC/3C6<br>/	AGATACG<br>G
LC92	GACGTGTGCTCTTCCGATCTNNNNNNNNNNNNNNTGAAGAGAGTCAC<br>CGTGTCGTCAATCC*T(25252525)	/5Phos/GGATTGACGACACGGTGAC/3C6<br>/	TGAAGAG<br>A
LC93	GACGTGTGCTCTTCCGATCTNNNNNNNNNNNNNNACGTCGTTGTCACC<br>GTGTCGTCAATCC*T(25252525)	/5Phos/GGATTGACGACACGGTGAC/3C6<br>/	ACGTCGTT
LC94	GACGTGTGCTCTTCCGATCTNNNNNNNNNNNNNNTGGCTTCAGTCACC<br>GTGTCGTCAATCC*T(25252525)	/5Phos/GGATTGACGACACGGTGAC/3C6<br>/	TGGCTTCA
LC95	GACGTGTGCTCTTCCGATCTNNNNNNNNNNNNNNGACATCTCGTCACC<br>GTGTCGTCAATCC*T(25252525)	/5Phos/GGATTGACGACACGGTGAC/3C6<br>/	GACATCTC
LC96	GACGTGTGCTCTTCCGATCTNNNNNNNNNNNNNNTTCACGCAGTCACC<br>GTGTCGTCAATCC*T(25252525)	/5Phos/GGATTGACGACACGGTGAC/3C6<br>/	TTCACGCA

PCR primers

Primer Name	Primer sequence
SK-LTRIII	AGCTTGCCTTGAGTGCTTCA
FB-Linker-P7	CAAGCAGAAGACGGCATACGAGATGTGACTGGAGTTCAGACGTGTGCTCTTCCGATC
P7	CAAGCAGAAGACGGCATACGAGAT

Name	Sequence (5'-3')	Barcode
LTR1	AATGATACGGCGACCACCGAGATCTACACTCTTTCCCTACACGACGCTCTTCCGATCTNNNNNNNNNNNNAA<br>ACATCGACCCTTTTAGTCAGTGTGGA	AAACATCG
LTR2	AATGATACGGCGACCACCGAGATCTACACTCTTTCCCTACACGACGCTCTTCCGATCTNNNNNNNNNNNNTG<br>CACTTGACCCTTTTAGTCAGTGTGGA	TGCACTTG
LTR3	AATGATACGGCGACCACCGAGATCTACACTCTTTCCCTACACGACGCTCTTCCGATCTNNNNNNNNNNNNAA<br>CCGAGAACCCTTTTAGTCAGTGTGGA	AACCGAGA
LTR4	AATGATACGGCGACCACCGAGATCTACACTCTTTCCCTACACGACGCTCTTCCGATCTNNNNNNNNNNNNCT<br>GATGAGACCCTTTTAGTCAGTGTGGA	CTGATGAG
LTR5	AATGATACGGCGACCACCGAGATCTACACTCTTTCCCTACACGACGCTCTTCCGATCTNNNNNNNNNNNNAA<br>CGTGATACCCTTTTAGTCAGTGTGGA	AACGTGAT
LTR6	AATGATACGGCGACCACCGAGATCTACACTCTTTCCCTACACGACGCTCTTCCGATCTNNNNNNNNNNNNG<br>AACGAAGACCCTTTTAGTCAGTGTGGA	GAACGAAG
LTR7	AATGATACGGCGACCACCGAGATCTACACTCTTTCCCTACACGACGCTCTTCCGATCTNNNNNNNNNNNNAA<br>GACGGAACCCTTTTAGTCAGTGTGGA	AAGACGGA
LTR8	AATGATACGGCGACCACCGAGATCTACACTCTTTCCCTACACGACGCTCTTCCGATCTNNNNNNNNNNNNGT<br>TGCTGTACCCTTTTAGTCAGTGTGGA	GTTGCTGT
LTR9	AATGATACGGCGACCACCGAGATCTACACTCTTTCCCTACACGACGCTCTTCCGATCTNNNNNNNNNNNNAA<br>GGACACACCCTTTTAGTCAGTGTGGA	AAGGACAC
LTR10	AATGATACGGCGACCACCGAGATCTACACTCTTTCCCTACACGACGCTCTTCCGATCTNNNNNNNNNNNNG<br>ACGAACTACCCTTTTAGTCAGTGTGGA	GACGAACT
LTR11	AATGATACGGCGACCACCGAGATCTACACTCTTTCCCTACACGACGCTCTTCCGATCTNNNNNNNNNNNNAA<br>TCCGTCACCCTTTTAGTCAGTGTGGA	AATCCGTC
LTR12	AATGATACGGCGACCACCGAGATCTACACTCTTTCCCTACACGACGCTCTTCCGATCTNNNNNNNNNNNNA<br>GGTAGGAACCCTTTTAGTCAGTGTGGA	AGGTAGGA
LTR13	AATGATACGGCGACCACCGAGATCTACACTCTTTCCCTACACGACGCTCTTCCGATCTNNNNNNNNNNNNAC<br>AAGCTAACCCTTTTAGTCAGTGTGGA	ACAAGCTA
LTR14	AATGATACGGCGACCACCGAGATCTACACTCTTTCCCTACACGACGCTCTTCCGATCTNNNNNNNNNNNNAC<br>TGGTGTACCCTTTTAGTCAGTGTGGA	ACTGGTGT
LTR15	AATGATACGGCGACCACCGAGATCTACACTCTTTCCCTACACGACGCTCTTCCGATCTNNNNNNNNNNNNAC<br>ACGACCACCCTTTTAGTCAGTGTGGA	ACACGACC
LTR16	AATGATACGGCGACCACCGAGATCTACACTCTTTCCCTACACGACGCTCTTCCGATCTNNNNNNNNNNNNA<br>GGCAATGACCCTTTTAGTCAGTGTGGA	AGGCAATG
LTR17	AATGATACGGCGACCACCGAGATCTACACTCTTTCCCTACACGACGCTCTTCCGATCTNNNNNNNNNNNNAC<br>AGCAGAACCCTTTTAGTCAGTGTGGA	ACAGCAGA
LTR18	AATGATACGGCGACCACCGAGATCTACACTCTTTCCCTACACGACGCTCTTCCGATCTNNNNNNNNNNNNTC<br>ACGATGACCCTTTTAGTCAGTGTGGA	TCACGATG
LTR19	AATGATACGGCGACCACCGAGATCTACACTCTTTCCCTACACGACGCTCTTCCGATCTNNNNNNNNNNNNAC<br>CACTGTACCCTTTTAGTCAGTGTGGA	ACCACTGT
LTR20	AATGATACGGCGACCACCGAGATCTACACTCTTTCCCTACACGACGCTCTTCCGATCTNNNNNNNNNNNNGC<br>AATGAGACCCTTTTAGTCAGTGTGGA	GCAATGAG
LTR21	AATGATACGGCGACCACCGAGATCTACACTCTTTCCCTACACGACGCTCTTCCGATCTNNNNNNNNNNNNAC<br>GCTCGAACCCTTTTAGTCAGTGTGGA	ACGCTCGA
LTR22	AATGATACGGCGACCACCGAGATCTACACTCTTTCCCTACACGACGCTCTTCCGATCTNNNNNNNNNNNNAT<br>CCGTTGACCCTTTTAGTCAGTGTGGA	ATCCGTTG
LTR23	AATGATACGGCGACCACCGAGATCTACACTCTTTCCCTACACGACGCTCTTCCGATCTNNNNNNNNNNNNAC<br>TATGCAACCCTTTTAGTCAGTGTGGA	ACTATGCA
LTR24	AATGATACGGCGACCACCGAGATCTACACTCTTTCCCTACACGACGCTCTTCCGATCTNNNNNNNNNNNNCG<br>TATCTCACCCTTTTAGTCAGTGTGGA	CGTATCTC
LTR25	AATGATACGGCGACCACCGAGATCTACACTCTTTCCCTACACGACGCTCTTCCGATCTNNNNNNNNNNNNA<br>GATCGCAACCCTTTTAGTCAGTGTGGA	AGATCGCA
LTR26	AATGATACGGCGACCACCGAGATCTACACTCTTTCCCTACACGACGCTCTTCCGATCTNNNNNNNNNNNNCC<br>GTTATGACCCTTTTAGTCAGTGTGGA	CCGTTATG
LTR27	AATGATACGGCGACCACCGAGATCTACACTCTTTCCCTACACGACGCTCTTCCGATCTNNNNNNNNNNNNA<br>GCACCTCACCCTTTTAGTCAGTGTGGA	AGCACCTC
LTR28	AATGATACGGCGACCACCGAGATCTACACTCTTTCCCTACACGACGCTCTTCCGATCTNNNNNNNNNNNNTA<br>GAACGCACCCTTTTAGTCAGTGTGGA	TAGAACGC
LTR29	AATGATACGGCGACCACCGAGATCTACACTCTTTCCCTACACGACGCTCTTCCGATCTNNNNNNNNNNNNA<br>GCCATGCACCCTTTTAGTCAGTGTGGA	AGCCATGC

Name	Sequence (5'-3')	Barcode
LTR30	AATGATACGGCGACCACCGAGATCTACACTCTTTCCCTACACGACGCTCTTCCGATCTNNNNNNNNNNNNACTCTGAGACCCTTTTAGTCAGTGTGGA	ACTCTGAG
LTR31	AATGATACGGCGACCACCGAGATCTACACTCTTTCCCTACACGACGCTCTTCCGATCTNNNNNNNNNNNNAGTACAAGACCCTTTTAGTCAGTGTGGA	AGTACAAG
LTR32	AATGATACGGCGACCACCGAGATCTACACTCTTTCCCTACACGACGCTCTTCCGATCTNNNNNNNNNNNNTGGCTCTTACCCTTTTAGTCAGTGTGGA	TGGCTCTT
LTR33	AATGATACGGCGACCACCGAGATCTACACTCTTTCCCTACACGACGCTCTTCCGATCTNNNNNNNNNNNNAGTGGTCAACCCTTTTAGTCAGTGTGGA	AGTGGTCA
LTR34	AATGATACGGCGACCACCGAGATCTACACTCTTTCCCTACACGACGCTCTTCCGATCTNNNNNNNNNNNNCCGTAACTACCCTTTTAGTCAGTGTGGA	CCGTAACT
LTR35	AATGATACGGCGACCACCGAGATCTACACTCTTTCCCTACACGACGCTCTTCCGATCTNNNNNNNNNNNNATCATTCCACCCTTTTAGTCAGTGTGGA	ATCATTCC
LTR36	AATGATACGGCGACCACCGAGATCTACACTCTTTCCCTACACGACGCTCTTCCGATCTNNNNNNNNNNNNGTCATCGTACCCTTTTAGTCAGTGTGGA	GTCATCGT
LTR37	AATGATACGGCGACCACCGAGATCTACACTCTTTCCCTACACGACGCTCTTCCGATCTNNNNNNNNNNNNATGCCTAAACCCTTTTAGTCAGTGTGGA	ATGCCTAA
LTR38	AATGATACGGCGACCACCGAGATCTACACTCTTTCCCTACACGACGCTCTTCCGATCTNNNNNNNNNNNNAGTGACCTACCCTTTTAGTCAGTGTGGA	AGTGACCT
LTR39	AATGATACGGCGACCACCGAGATCTACACTCTTTCCCTACACGACGCTCTTCCGATCTNNNNNNNNNNNNATTGGCTCACCCTTTTAGTCAGTGTGGA	ATTGGCTC
LTR40	AATGATACGGCGACCACCGAGATCTACACTCTTTCCCTACACGACGCTCTTCCGATCTNNNNNNNNNNNNTCAGTAGGACCCTTTTAGTCAGTGTGGA	TCAGTAGG
LTR41	AATGATACGGCGACCACCGAGATCTACACTCTTTCCCTACACGACGCTCTTCCGATCTNNNNNNNNNNNNCAAGACTAACCCTTTTAGTCAGTGTGGA	CAAGACTA
LTR42	AATGATACGGCGACCACCGAGATCTACACTCTTTCCCTACACGACGCTCTTCCGATCTNNNNNNNNNNNNGCGCATATACCCTTTTAGTCAGTGTGGA	GCGCATAT
LTR43	AATGATACGGCGACCACCGAGATCTACACTCTTTCCCTACACGACGCTCTTCCGATCTNNNNNNNNNNNNCAATGGAAACCCTTTTAGTCAGTGTGGA	CAATGGAA
LTR44	AATGATACGGCGACCACCGAGATCTACACTCTTTCCCTACACGACGCTCTTCCGATCTNNNNNNNNNNNNTAGGAGCTACCCTTTTAGTCAGTGTGGA	TAGGAGCT
LTR45	AATGATACGGCGACCACCGAGATCTACACTCTTTCCCTACACGACGCTCTTCCGATCTNNNNNNNNNNNNCACTTCGAACCCTTTTAGTCAGTGTGGA	CACTTCGA
LTR46	AATGATACGGCGACCACCGAGATCTACACTCTTTCCCTACACGACGCTCTTCCGATCTNNNNNNNNNNNNTCAATCCGACCCTTTTAGTCAGTGTGGA	TCAATCCG
LTR47	AATGATACGGCGACCACCGAGATCTACACTCTTTCCCTACACGACGCTCTTCCGATCTNNNNNNNNNNNNCAGCGTTAACCCTTTTAGTCAGTGTGGA	CAGCGTTA
LTR48	AATGATACGGCGACCACCGAGATCTACACTCTTTCCCTACACGACGCTCTTCCGATCTNNNNNNNNNNNNCTTCCTTCACCCTTTTAGTCAGTGTGGA	CTTCCTTC
LTR49	AATGATACGGCGACCACCGAGATCTACACTCTTTCCCTACACGACGCTCTTCCGATCTNNNNNNNNNNNNCATCAAGTACCCTTTTAGTCAGTGTGGA	CATCAAGT
LTR50	AATGATACGGCGACCACCGAGATCTACACTCTTTCCCTACACGACGCTCTTCCGATCTNNNNNNNNNNNNATAGTCGGACCCTTTTAGTCAGTGTGGA	ATAGTCGG
LTR51	AATGATACGGCGACCACCGAGATCTACACTCTTTCCCTACACGACGCTCTTCCGATCTNNNNNNNNNNNNCCATCCTCACCCTTTTAGTCAGTGTGGA	CCATCCTC
LTR52	AATGATACGGCGACCACCGAGATCTACACTCTTTCCCTACACGACGCTCTTCCGATCTNNNNNNNNNNNNCTTACAGCACCCTTTTAGTCAGTGTGGA	CTTACAGC
LTR53	AATGATACGGCGACCACCGAGATCTACACTCTTTCCCTACACGACGCTCTTCCGATCTNNNNNNNNNNNNCCGACAACACCCTTTTAGTCAGTGTGGA	CCGACAAC
LTR54	AATGATACGGCGACCACCGAGATCTACACTCTTTCCCTACACGACGCTCTTCCGATCTNNNNNNNNNNNNTCGAACCTACCCTTTTAGTCAGTGTGGA	TCGAACCT
LTR55	AATGATACGGCGACCACCGAGATCTACACTCTTTCCCTACACGACGCTCTTCCGATCTNNNNNNNNNNNNCCTAATCCACCCTTTTAGTCAGTGTGGA	CCTAATCC
LTR56	AATGATACGGCGACCACCGAGATCTACACTCTTTCCCTACACGACGCTCTTCCGATCTNNNNNNNNNNNNGCTGTAAGACCCTTTTAGTCAGTGTGGA	GCTGTAAG
LTR57	AATGATACGGCGACCACCGAGATCTACACTCTTTCCCTACACGACGCTCTTCCGATCTNNNNNNNNNNNNCCTCTATCACCCTTTTAGTCAGTGTGGA	CCTCTATC
LTR58	AATGATACGGCGACCACCGAGATCTACACTCTTTCCCTACACGACGCTCTTCCGATCTNNNNNNNNNNNNCTCGTTCTACCCTTTTAGTCAGTGTGGA	CTCGTTCT
LTR59	AATGATACGGCGACCACCGAGATCTACACTCTTTCCCTACACGACGCTCTTCCGATCTNNNNNNNNNNNNCGACACACACCCTTTTAGTCAGTGTGGA	CGACACAC
LTR60	AATGATACGGCGACCACCGAGATCTACACTCTTTCCCTACACGACGCTCTTCCGATCTNNNNNNNNNNNNAACAGGTGACCCTTTTAGTCAGTGTGGA	AACAGGTG
LTR61	AATGATACGGCGACCACCGAGATCTACACTCTTTCCCTACACGACGCTCTTCCGATCTNNNNNNNNNNNNCGCATACAACCCTTTTAGTCAGTGTGGA	CGCATACA
LTR62	AATGATACGGCGACCACCGAGATCTACACTCTTTCCCTACACGACGCTCTTCCGATCTNNNNNNNNNNNNTTCGCCATACCCTTTTAGTCAGTGTGGA	TTCGCCAT
LTR63	AATGATACGGCGACCACCGAGATCTACACTCTTTCCCTACACGACGCTCTTCCGATCTNNNNNNNNNNNNCGGATTGCACCCTTTTAGTCAGTGTGGA	CGGATTGC

Name	Sequence (5'-3')	Barcode
LTR64	AATGATACGGCGACCACCGAGATCTACACTCTTTCCCTACACGACGCTCTTCCGATCTNNNNNNNNNNNNATAGAGCGACCCTTTTAGTCAGTGTGGA	ATAGAGCG
LTR65	AATGATACGGCGACCACCGAGATCTACACTCTTTCCCTACACGACGCTCTTCCGATCTNNNNNNNNNNNNCTCAATGAACCCTTTTAGTCAGTGTGGA	CTCAATGA
LTR66	AATGATACGGCGACCACCGAGATCTACACTCTTTCCCTACACGACGCTCTTCCGATCTNNNNNNNNNNNNCTGAACGTACCCTTTTAGTCAGTGTGGA	CTGAACGT
LTR67	AATGATACGGCGACCACCGAGATCTACACTCTTTCCCTACACGACGCTCTTCCGATCTNNNNNNNNNNNNCTGGCATAACCCTTTTAGTCAGTGTGGA	CTGGCATA
LTR68	AATGATACGGCGACCACCGAGATCTACACTCTTTCCCTACACGACGCTCTTCCGATCTNNNNNNNNNNNNTGATCACGACCCTTTTAGTCAGTGTGGA	TGATCACG
LTR69	AATGATACGGCGACCACCGAGATCTACACTCTTTCCCTACACGACGCTCTTCCGATCTNNNNNNNNNNNNGAACAGGCACCCTTTTAGTCAGTGTGGA	GAACAGGC
LTR70	AATGATACGGCGACCACCGAGATCTACACTCTTTCCCTACACGACGCTCTTCCGATCTNNNNNNNNNNNNCATACTCGACCCTTTTAGTCAGTGTGGA	CATACTCG
LTR71	AATGATACGGCGACCACCGAGATCTACACTCTTTCCCTACACGACGCTCTTCCGATCTNNNNNNNNNNNNGACAGTGCACCCTTTTAGTCAGTGTGGA	GACAGTGC
LTR72	AATGATACGGCGACCACCGAGATCTACACTCTTTCCCTACACGACGCTCTTCCGATCTNNNNNNNNNNNNAGTGCATCACCCTTTTAGTCAGTGTGGA	AGTGCATC
LTR73	AATGATACGGCGACCACCGAGATCTACACTCTTTCCCTACACGACGCTCTTCCGATCTNNNNNNNNNNNNGAGCTGAAACCCTTTTAGTCAGTGTGGA	GAGCTGAA
LTR74	AATGATACGGCGACCACCGAGATCTACACTCTTTCCCTACACGACGCTCTTCCGATCTNNNNNNNNNNNNCTAGCAGTACCCTTTTAGTCAGTGTGGA	CTAGCAGT
LTR75	AATGATACGGCGACCACCGAGATCTACACTCTTTCCCTACACGACGCTCTTCCGATCTNNNNNNNNNNNNGATAGACAACCCTTTTAGTCAGTGTGGA	GATAGACA
LTR76	AATGATACGGCGACCACCGAGATCTACACTCTTTCCCTACACGACGCTCTTCCGATCTNNNNNNNNNNNNTTCGTACGACCCTTTTAGTCAGTGTGGA	TTCGTACG
LTR77	AATGATACGGCGACCACCGAGATCTACACTCTTTCCCTACACGACGCTCTTCCGATCTNNNNNNNNNNNNGCCAAGACACCCTTTTAGTCAGTGTGGA	GCCAAGAC
LTR78	AATGATACGGCGACCACCGAGATCTACACTCTTTCCCTACACGACGCTCTTCCGATCTNNNNNNNNNNNNTGTCGACTACCCTTTTAGTCAGTGTGGA	TGTCGACT
LTR79	AATGATACGGCGACCACCGAGATCTACACTCTTTCCCTACACGACGCTCTTCCGATCTNNNNNNNNNNNNGCGAGTAAACCCTTTTAGTCAGTGTGGA	GCGAGTAA
LTR80	AATGATACGGCGACCACCGAGATCTACACTCTTTCCCTACACGACGCTCTTCCGATCTNNNNNNNNNNNNCAGTGCTTACCCTTTTAGTCAGTGTGGA	CAGTGCTT
LTR81	AATGATACGGCGACCACCGAGATCTACACTCTTTCCCTACACGACGCTCTTCCGATCTNNNNNNNNNNNNGCTCGGTAACCCTTTTAGTCAGTGTGGA	GCTCGGTA
LTR82	AATGATACGGCGACCACCGAGATCTACACTCTTTCCCTACACGACGCTCTTCCGATCTNNNNNNNNNNNNAAGGAAGGACCCTTTTAGTCAGTGTGGA	AAGGAAGG
LTR83	AATGATACGGCGACCACCGAGATCTACACTCTTTCCCTACACGACGCTCTTCCGATCTNNNNNNNNNNNNGGTGCGAAACCCTTTTAGTCAGTGTGGA	GGTGCGAA
LTR84	AATGATACGGCGACCACCGAGATCTACACTCTTTCCCTACACGACGCTCTTCCGATCTNNNNNNNNNNNNGATCCACTACCCTTTTAGTCAGTGTGGA	GATCCACT
LTR85	AATGATACGGCGACCACCGAGATCTACACTCTTTCCCTACACGACGCTCTTCCGATCTNNNNNNNNNNNNGTCGTAGAACCCTTTTAGTCAGTGTGGA	GTCGTAGA
LTR86	AATGATACGGCGACCACCGAGATCTACACTCTTTCCCTACACGACGCTCTTCCGATCTNNNNNNNNNNNNCCTTAGGTACCCTTTTAGTCAGTGTGGA	CCTTAGGT
LTR87	AATGATACGGCGACCACCGAGATCTACACTCTTTCCCTACACGACGCTCTTCCGATCTNNNNNNNNNNNNGTGTTCTAACCCTTTTAGTCAGTGTGGA	GTGTTCTA
LTR88	AATGATACGGCGACCACCGAGATCTACACTCTTTCCCTACACGACGCTCTTCCGATCTNNNNNNNNNNNNGTATTCCGACCCTTTTAGTCAGTGTGGA	GTATTCCG
LTR89	AATGATACGGCGACCACCGAGATCTACACTCTTTCCCTACACGACGCTCTTCCGATCTNNNNNNNNNNNNTATCAGCAACCCTTTTAGTCAGTGTGGA	TATCAGCA
LTR90	AATGATACGGCGACCACCGAGATCTACACTCTTTCCCTACACGACGCTCTTCCGATCTNNNNNNNNNNNNAGGTCTGTACCCTTTTAGTCAGTGTGGA	AGGTCTGT
LTR91	AATGATACGGCGACCACCGAGATCTACACTCTTTCCCTACACGACGCTCTTCCGATCTNNNNNNNNNNNNTCTTCACAACCCTTTTAGTCAGTGTGGA	TCTTCACA
LTR92	AATGATACGGCGACCACCGAGATCTACACTCTTTCCCTACACGACGCTCTTCCGATCTNNNNNNNNNNNNGATGTCGAACCCTTTTAGTCAGTGTGGA	GATGTCGA
LTR93	AATGATACGGCGACCACCGAGATCTACACTCTTTCCCTACACGACGCTCTTCCGATCTNNNNNNNNNNNNTGGAACAAACCCTTTTAGTCAGTGTGGA	TGGAACAA
LTR94	AATGATACGGCGACCACCGAGATCTACACTCTTTCCCTACACGACGCTCTTCCGATCTNNNNNNNNNNNNTACACACGACCCTTTTAGTCAGTGTGGA	TACACACG
LTR95	AATGATACGGCGACCACCGAGATCTACACTCTTTCCCTACACGACGCTCTTCCGATCTNNNNNNNNNNNNTGGTGGTAACCCTTTTAGTCAGTGTGGA	TGGTGGTA
LTR96	AATGATACGGCGACCACCGAGATCTACACTCTTTCCCTACACGACGCTCTTCCGATCTNNNNNNNNNNNNGACACAGTACCCTTTTAGTCAGTGTGGA	GACACAGT

References for Supplementary Notes

1 Lance, G. N. & Williams, W. T. Computer Programs for Hierarchical Polythetic Classification ("Similarity Analyses"). *The Computer Journal* **9**, 60-64, doi:10.1093/comjnl/9.1.60 (1966).

2 Kaufman, L. & Rousseeuw, P. J. in *Finding Groups in Data Wiley Series in Probability and Statistics* 68-125 (1990).

3 Rousseeuw, P. J. Silhouettes: A graphical aid to the interpretation and validation of cluster analysis. *Journal of Computational and Applied Mathematics* **20**, 53-65, doi:10.1016/0377-0427(87)90125-7 (1987).

4 Baldow, C., Thielecke, L. & Glauche, I. Model Based Analysis of Clonal Developments Allows for Early Detection of Monoclonal Conversion and Leukemia. *PLoS One* **11**, e0165129, doi:10.1371/journal.pone.0165129 (2016).

5 Montini, E. *et al.* The genotoxic potential of retroviral vectors is strongly modulated by vector design and integration site selection in a mouse model of HSC gene therapy. *J Clin Invest* **119**, 964-975, doi:10.1172/JCI37630 (2009).

6 Montini, E. *et al.* Hematopoietic stem cell gene transfer in a tumor-prone mouse model uncovers low genotoxicity of lentiviral vector integration. *Nat Biotechnol* **24**, 687-696, doi:10.1038/nbt1216 (2006).

7 Firouzi, S. *et al.* Development and validation of a new high-throughput method to investigate the clonality of HTLV-1-infected cells based on provirus integration sites. *Genome Medicine* **6**, 46, doi:10.1186/gm568 (2014).

8 Schmidt, M. *et al.* High-resolution insertion-site analysis by linear amplification–mediated PCR (LAM-PCR). *Nature Methods* **4**, 1051-1057, doi:10.1038/nmeth1103 (2007).

9 Cesana, D. *et al.* Retrieval of vector integration sites from cell-free DNA. *Nat Med* **27**, 1458-1470, doi:10.1038/s41591-021-01389-4 (2021).

10 Spinozzi, G. *et al.* VISPA2: a scalable pipeline for high-throughput identification and annotation of vector integration sites. *BMC Bioinformatics* **18**, doi:10.1186/s12859-017-1937-9 (2017).

11 Berry, C. C. *et al.* Estimating Abundances of Retroviral Insertion Sites from DNA Fragment Length Data. *Bioinformatics (Oxford, England)*, 1-8, doi:10.1093/bioinformatics/bts004 (2012).

12 Espinoza, D. A. *et al.* Aberrant Clonal Hematopoiesis following Lentiviral Vector Transduction of HSPCs in a Rhesus Macaque. *Molecular Therapy* **27**, 1074-1086, doi:10.1016/j.ymthe.2019.04.003 (2019).

13 Halkidi, M., Vazirgiannis, M. & Batistakis, Y. 265-276 (Springer Berlin Heidelberg).

14 Kraemer, H. Biserial Correlation. **1**, 276-279, doi:10.1002/0471667196.ess0153.pub2 (2006).

15 Milligan, G. W. An examination of the effect of six types of error perturbation on fifteen clustering algorithms. *Psychometrika* **45**, 325-342, doi:10.1007/bf02293907 (1980).

16 Milligan, G. W. A monte carlo study of thirty internal criterion measures for cluster analysis. *Psychometrika* **46**, 187-199, doi:10.1007/bf02293899 (1981).

17 Dunn†, J. C. Well-Separated Clusters and Optimal Fuzzy Partitions. *Journal of Cybernetics* **4**, 95-104, doi:10.1080/01969727408546059 (2008).
