# Supplementary material for: Clonal reconstruction from co-occurrence of vector integration sites accurately quantifies expanding clones in vivo
Source: Nat Commun. 2022 Jun 28;13:3712. doi: 10.1038/s41467-022-31292-6 (PMC9240075; doi:10.1038/s41467-022-31292-6)
Supplement: Supplementary file 2 — Reporting Summary [file 41467_2022_31292_MOESM2_ESM.pdf]

nature research

Corresponding author(s): Ingmar Glauche

Last updated by author(s): Jun 7, 2022

Reporting Summary

Nature Research wishes to improve the reproducibility of the work that we publish. This form provides structure for consistency and transparency in reporting. For further information on Nature Research policies, see our Editorial Policies and the Editorial Policy Checklist.

Statistics

For all statistical analyses, confirm that the following items are present in the figure legend, table legend, main text, or Methods section.

n/a	Confirmed	
☐	☒	The exact sample size (*n*) for each experimental group/condition, given as a discrete number and unit of measurement
☒	☐	A statement on whether measurements were taken from distinct samples or whether the same sample was measured repeatedly
☒	☐	The statistical test(s) used AND whether they are one- or two-sided<br>*Only common tests should be described solely by name; describe more complex techniques in the Methods section.*
☒	☐	A description of all covariates tested
☒	☐	A description of any assumptions or corrections, such as tests of normality and adjustment for multiple comparisons
☐	☒	A full description of the statistical parameters including central tendency (e.g. means) or other basic estimates (e.g. regression coefficient) AND variation (e.g. standard deviation) or associated estimates of uncertainty (e.g. confidence intervals)
☒	☐	For null hypothesis testing, the test statistic (e.g. *F*, *t*, *r*) with confidence intervals, effect sizes, degrees of freedom and *P* value noted<br>*Give P values as exact values whenever suitable.*
☒	☐	For Bayesian analysis, information on the choice of priors and Markov chain Monte Carlo settings
☒	☐	For hierarchical and complex designs, identification of the appropriate level for tests and full reporting of outcomes
☒	☐	Estimates of effect sizes (e.g. Cohen's *d*, Pearson's *r*), indicating how they were calculated

Our web collection on statistics for biologists contains articles on many of the points above.

Software and code

Policy information about availability of computer code

Data collection	Data were generated using experimental procedures described in the manuscript. Sequencing reads for the validation assay dataset and genotoxic experiment in mice were generated with Illumina paired-end reads and processed by a dedicated bioinformatics pipeline (VISPA2) as previously described in Spinozzi et al, BMC Bioinformatics, 2017. Additional specification are reported in the Supplementary Information file. We use QuantaSoft Version 1.7.4.0917 (Biorad) to analyze ddPCR data.
Data analysis	We used the statistical functions from the "base" and the "stats" packages in R version 4.0.5 to compute basic descriptive statistics. Custom R code, uploaded in the GitLab repository, has been used to analyze data and is available at https://gitlab.com/imb-dev/clonal-reconstruction-figures/.<br>The main software MultIS 0.6.2 is released in the CRAN repository and all paper figures/data are available in the GitLab repository (https://gitlab.com/imb-dev/clonal-reconstruction-figures/). We used the PAM implementation "pam" from version 2.1.2 of the "cluster" package for R.

For manuscripts utilizing custom algorithms or software that are central to the research but not yet described in published literature, software must be made available to editors and reviewers. We strongly encourage code deposition in a community repository (e.g. GitHub). See the Nature Research guidelines for submitting code & software for further information.

Data

Policy information about availability of data

All manuscripts must include a data availability statement. This statement should provide the following information, where applicable:

- Accession codes, unique identifiers, or web links for publicly available datasets
- A list of figures that have associated raw data
- A description of any restrictions on data availability

All datasets analyzed within the current study are available from the clonal-reconstruction-figures repository at https://gitlab.com/imb-dev/clonal-reconstruction-figures/-/tree/master/data. The macaque data set can be obtained from (Espinoza et al., 2019).

Field-specific reporting

Please select the one below that is the best fit for your research. If you are not sure, read the appropriate sections before making your selection.

☒ Life sciences ☐ Behavioural & social sciences ☐ Ecological, evolutionary & environmental sciences

For a reference copy of the document with all sections, see nature.com/documents/nr-reporting-summary-flat.pdf

Life sciences study design

All studies must disclose on these points even when the disclosure is negative.

Sample size	In the mouse study, we used tumor prone mice Cdkn2a -/- and treated neutral vector (SIN.LV.PGK.GFP.PRE). Given that experimental results in mice have been already described in literature (Montini et al, Nat. Biotech, 2006; Montini, Cesana et al, JCI, 2009), we used a subset of mice of an experimental study that originally calculated sample size to identify differences in survival at 255 days post treatment between Cdkn2a -/- and treated with genotoxic vector (LV.SF.LTR) or neutral vector (SIN.LV.PGK.GFP.PRE); sample power of 0.8 and alpha level of 0.05.
Data exclusions	No exclusions have been applied a priori.
Replication	The experimental results in mice have been already described in literature (Montini et al, Nat. Biotech, 2006; Montini, Cesana et al, JCI, 2009). Our current data set is considered as a successful experimental replication.
Randomization	The experimental design did not include allocation of samples to randomized experimental group since we replicated the experimental results reported in Montini et al, Nat. Biotech 2006 to analyze vector integration sites. All mice were kept under identical conditions to control for related covariates.
Blinding	Blinding in the study was not necessary as the relevant subcohort of mice was treated equally and kept under identical conditions.

Reporting for specific materials, systems and methods

We require information from authors about some types of materials, experimental systems and methods used in many studies. Here, indicate whether each material, system or method listed is relevant to your study. If you are not sure if a list item applies to your research, read the appropriate section before selecting a response.

Materials & experimental systems

n/a	Involved in the study	
☒	☐	Antibodies
☐	☒	Eukaryotic cell lines
☒	☐	Palaeontology and archaeology
☐	☒	Animals and other organisms
☒	☐	Human research participants
☒	☐	Clinical data
☒	☐	Dual use research of concern

Methods

n/a	Involved in the study	
☒	☐	ChIP-seq
☐	☒	Flow cytometry
☒	☐	MRI-based neuroimaging

Eukaryotic cell lines

Policy information about cell lines

Cell line source(s)	k-562 (K562 cells are an erythroleukemia cell type derived from a 53-year-old female chronic myelogenous leukemia patient in blast crisis) and Jy cell line (an Epstein–Barr virus (EBV)-immortalised B cell lymphoblastoid line) were available in the lab
Authentication	None of the cell line used were authenticated
Mycoplasma contamination	The cell lines adopted were negative for the presence of mycoplasma. Mycoplasma is detected by specific- assay, MycoAlert Plus Mycoplasma (Lonza, LT707-710)
Commonly misidentified lines (See ICLAC register)	None

Animals and other organisms

Policy information about studies involving animals; ARRIVE guidelines recommended for reporting animal research

Laboratory animals	C57BL6/J-Cdkn2a-/- mice were obtained from the National Cancer Institute Mouse Models of Human Cancer Consortium. Lin- cells were collected from n=12 Cdkn2a-/- donor mice, half female and half male, aged 8 weeks. Transduced cells were transplanted in 6-8 female, lethally irradiated wild-type C57BL6/J mice (aged 8 weeks). Wild-type C57BL6/J mice were obtained by Charles River Laboratories.
Wild animals	No wild animals were used in this study.
Field-collected samples	The study did not involve field collected samples.
Ethics oversight	All procedures were performed according to protocols approved by the Animal Care and Use Committee of the San Raffaele Institute (IACUC 859) and communicated to the Ministry of Health and local authorities according to Italian law.

Note that full information on the approval of the study protocol must also be provided in the manuscript.

Flow Cytometry

Plots

Confirm that:

- [x] The axis labels state the marker and fluorochrome used (e.g. CD4-FITC).
- [x] The axis scales are clearly visible. Include numbers along axes only for bottom left plot of group (a 'group' is an analysis of identical markers).
- [x] All plots are contour plots with outliers or pseudocolor plots.
- [x] A numerical value for number of cells or percentage (with statistics) is provided.

Methodology

Sample preparation	Bleedings were performed approximately every 4 weeks starting 4 weeks after transplantation. Prior to bleeding procedures mice were anesthetized via isoflurane inhalation. Thymus, BM and spleen tissues wereharvested at euthanasia for phenotypic analyses.Peripheral blood were collected from transplanted mice via retro-orbital bleeding using heparinized capillary glass tubes. Total blood were stained in FACS polystyrene tubes with a 1:100 ratio of each Ab and incubated in the dark for 20 minutes at room temperature. Each sample was then divided into aliquots where 100µl of FBS were added to each aliquot to reach the optimal protein concentration for red blood cell lysis using the automated red blood cell lysis system TQ-Prep Workstation and IMMUNOPREP Reagent System (7546946) from Beckman Coulter. Lymphoid B and T cells as well as myeloid cells were isolated by fluorescence activated cell sorting using Lineage-specific antibodies (Abs) against murine CD11b (myeloid marker), CD19 (B-lymphoid marker), CD3 (T-lymphoid marker). Antibodies used were purchased from BD Biosciences Pharmingen: rat anti-mouse CD11b (BV421); rat anti-mouse CD3 (PE); rat anti-mouse CD19 (APC). To recover enough DNA material, equal amounts of blood from two or three mice belonging to the same experimental group were pooled before the sorting procedure. The composition of pools was maintained constant throughout the whole experiment, so that each pool is composed by the same mice over time.
Instrument	Flow cytometry analysis were conducted using FACS Canto (Becton Dickinson Pharmingen). Flow sorting was conducted using FACS Moflo XDP (Beckman Coulter)
Software	Flow cytometry data was analyzed by FCS express.
Cell population abundance	A minimum of 30000 events have been acquired for each sample.

Gating strategy

In all Flow Cytometry and cell sorting procedures single cells were selected by representing FSC Height/Weight parameter vs Area. Then, cell populations were selected based on their FSC-A/SSC-A parameter. Cells expressing the mentioned markers were gated based on the respective negative controls of unstained cells, simple positive controls and SSC-A/FSC-A profiles to ensure the population.

[x] Tick this box to confirm that a figure exemplifying the gating strategy is provided in the Supplementary Information.
